# Impact of Population Pharmacogenomics on Cisplatin‐Induced Neurotoxicities in Testicular Cancer Survivors

**DOI:** 10.1002/cam4.71218

**Published:** 2025-09-05

**Authors:** Swetha Nakshatri, Paul C. Dinh, Lawrence H. Einhorn, Darren R. Feldman, Robert J. Hamilton, David J. Vaughn, Chunkit Fung, Christian Kollmannsberger, Robert A. Huddart, Lois B. Travis, Nancy J. Cox, M. Eileen Dolan

**Affiliations:** ^1^ Department of Medicine University of Chicago Chicago Illinois USA; ^2^ Department of Medical Oncology Indiana University Indianapolis Indiana USA; ^3^ Department of Medical Oncology Memorial Sloan‐Kettering Cancer Center New York New York USA; ^4^ Department of Surgical Oncology Princess Margaret Cancer Centre Toronto Ontario Canada; ^5^ Department of Medicine University of Pennsylvania Philadelphia Pennsylvania USA; ^6^ J.P. Wilmot Cancer Institute University of Rochester Medical Center Rochester New York USA; ^7^ Division of Medical Oncology University of British Columbia Vancouver British Columbia Canada; ^8^ Royal Marsden Hospital London UK; ^9^ Department of Epidemiology, Fairbanks School of Public Health Indiana University Indianapolis Indiana USA; ^10^ Department of Medicine Vanderbilt University Medical Center Nashville Tennessee USA

**Keywords:** cisplatin, disparities, genetic variants, neurotoxicity, pharmacogenomics, vertigo

## Abstract

**Background:**

Cisplatin is a commonly used chemotherapeutic across numerous cancer types that can cause neurotoxicities in patients, including peripheral sensory neuropathy, tinnitus, hearing loss, and vertigo.

**Objective:**

We aimed to evaluate, for the first time, how genetic ancestry impacts cisplatin‐induced neurotoxicities and if disparities are related to population differences in allele frequency.

**Methods:**

In a cohort of cisplatin‐treated testicular cancer survivors, relationships between genetic ancestry and neurotoxicities, medications, and lifestyle factors were assessed using logistic regression and Kruskal–Wallis tests and multiple pairwise comparisons using the Wilcoxon rank‐sum test (Benjamini–Hochberg adjustment). Associations between single nucleotide polymorphism (SNP) genotypes and neurotoxicities with significant inter‐population disparities were calculated to identify independent, functional variants with population allele frequency differentiation associated with toxicities.

**Results:**

Following four cycles of cisplatin‐based chemotherapy, African ancestry survivors were significantly more likely to have neuropathy and vertigo versus European and Asian‐axis ancestry survivors, although Asian axis survivors were significantly younger at evaluation than other ancestries. Following filtering for population allele frequency differentiation, functional relevance, and independence, 19,992 SNPs were tested for association with toxicities. Although none passed the Bonferroni threshold, two and four SNPs were associated with neuropathy and vertigo, respectively, at suggestively significant *p* < 1.0 × 10^−4^. For neuropathy, rs34904346 (*p* = 2.0 × 10^−5^) was an expression quantitative trait locus (eQTL) for RNF24 in nerve tissue, with three other RNF24 eQTLs associated with neuropathy (*p* < 0.01). For vertigo, rs3777909 (*p* = 3.1 × 10^−5^) was an eQTL for MFSD4B in nerve and REV3L in brain tissue, along with three other eQTLs for MFSD4B and four for REV3L associated with vertigo (*p* < 0.05). In silico, higher MFSD4B and REV3L expression in cancer cell lines were associated with significantly greater cisplatin sensitivity.

**Conclusion:**

African ancestry was associated with increased cisplatin‐induced peripheral sensory neuropathy and vertigo versus European ancestry. Population allele frequency differences and expression levels of RNF24, MFSD4B, and REV3L were potentially implicated.

## 1 Introduction

Advances in cancer screening and treatment have reduced mortality, leaving millions of survivors at risk for long‐term toxicities [1, 2]. Cisplatin, used to treat many cancers [3], can cause severe neurotoxicities, including peripheral sensory neuropathy (PSN), tinnitus, and hearing loss (HL) [4, 5]. Ototoxicity can create functional limitations, including sleep disorders, anxiety/depression, and cognitive decline [6, 7, 8, 9]. Cisplatin‐induced PSN can affect quality of life, with no effective treatments except for some benefit of duloxetine for those with painful neuropathy [10]. Identification of risk factors is critical to stratify patients for prevention and to develop effective treatments for toxicities.

Although studies have described population differences in paclitaxel and vincristine‐induced neurotoxicities [11, 12, 13, 14, 15], scarce data exists for cisplatin‐induced neurotoxicities. Breast cancer survivors of African (vs. European) ancestry have significantly greater paclitaxel‐induced neuropathy [11, 12, 13, 14]. In contrast, African American survivors of childhood acute lymphoblastic leukemia (ALL) given vincristine have less neurotoxicity than European Americans [15, 16]. Pharmacogenomic variants vary in allele frequency across ancestral populations [17, 18]. Cisplatin‐related vascular toxicity is similar to toxicity associated with environmental heavy metals, with their metabolism impacted by genetic variation, potentially related to adaptation to different environmental exposures across geographies [19, 20].

Given the deficit of data regarding population differences in cisplatin‐induced neurotoxicities, we evaluated testicular cancer survivors (TCS) treated with cisplatin‐based chemotherapy to assess how genetic ancestry impacts the prevalence and severity of cisplatin‐induced PSN, HL, tinnitus, and vertigo. Since race is a social construct, we used genetic ancestry and focused on functional ancestry‐informative SNP markers with significant allele frequency differentiation between populations.

## 2 Materials and Methods

### 2.1 Population

Survivors were enrolled in The Platinum Study, including eight institutions in the US, UK, and Canada. Eligibility criteria, previously described [21, 22], included men who were diagnosed with a germ cell tumor (histologically or serologically confirmed) before the age of 60, who were over 18 at the time of consent for the study, who received cisplatin treatment, and who had no follow‐up chemotherapy treatment. Informed written consent was obtained from all participants; protocols were approved by each institution's Human Subject Review Board. Procedures followed the U.S. Common Rule.

### 2.2 Toxicity Phenotypes

PSN was defined using eight relevant items in the European Organization for Research and Treatment of Cancer‐Chemotherapy‐Induced Peripheral Neuropathy‐20 (EORTC‐CIPN20) questionnaire [23]. Phenotypes included tingling in hands/feet, numbness in hands/feet, shooting or burning pain in hands/feet, problems standing or walking due to difficulty feeling the ground, and difficulty distinguishing between hot/cold water. Mean of responses was converted to a scale using the ceiling function: 0: “none”, 1: “a little” 2: “quite a bit”, 3: “very much.” Categories 2/3 were combined to define severe neuropathy and categories 1/2/3 were combined to define the any (“yes”) versus none (“no”) categories for overall neuropathy.

For HL, a summary measure was derived from audiometry by calculating the geometric mean of bilateral hearing thresholds from 4 to 12 kHz [21].

Tinnitus was based on: “In the last 4 weeks, have you had ringing or buzzing in the ears?” in the Scale for Chemotherapy‐induced Long‐term Neurotoxicity questionnaire [24]. Those responding “not at all” were controls, with “quite a bit” or “very much” defining cases; “a little” responses were excluded. Consistency was checked with a second question asked: “Do you have ringing or buzzing in the ears?”

Vertigo was defined by querying persistent dizziness or vertigo (yes/no/don't know‐not sure) with no for controls and yes for cases.

### 2.3 Questionnaires

Participants completed questionnaires regarding lifestyle behaviors, adverse health outcomes, and prescription medications [25]. Alcoholic drinks were queried on a scale from 0 to 8. Categories 6–8 (> 2 drinks/day) defined “excessive drinking.” Survivors were queried regarding current/past smoking, prescription medications (hypertension and/or hypercholesterolemia), and diabetes (with/without insulin or prescription medication). Answers were “never,” “past only,” “yes, current,” and “don't know/not sure.” “Never” responses defined controls; “yes, current” defined cases. Self‐reported health was evaluated with: “would you rate your health as: (1) excellent, (2) very good, (3) good, (4) fair, or (5) poor?” “Fair” and “poor” were combined, due to low numbers. For association analysis, “good”, “very good”, and “excellent” were combined.

### 2.4 Genotyping and Imputation

DNA from blood was genotyped using The Infinium Global Screening Array‐24 chip (GSA‐24v1‐0_A1; Illumina) at Regeneron Pharmaceuticals. Subject quality control (QC) excluded subjects with missingness > 0.2, followed by > 0.02, and included those with pairwise identity by descent < 0.2 and subjects within ±3 standard deviations (SD) of the mean heterozygosity rate. For pairs with identity by descent >0.2, the subject with the lower call rate (higher missing call rate) was removed. SNP QC excluded SNPs with missingness > 0.2, followed by > 0.02, minor allele frequency (MAF) < 0.05, and Hardy–Weinberg Equilibrium (HWE) *p* < 1 × 10^−6^. Sex discrepancies were assessed based on X chromosome homozygosity estimates. Imputation was conducted on the University of Michigan Imputation Server with the 1000 Genomes Phase 3 Reference Panel. Post‐imputation QC excluded SNPs with imputation *R* ^2^ < 0.8, MAF < 0.05, and HWE *p*‐value < 1 × 10^−6^ (Figure S1).

### 2.5 Genetic Ancestry

Ancestry was determined by multidimensional scaling. Scores were plotted and anchored by ancestry data from the 1000 Genomes population. Based on visual cutoffs, survivors were divided into European ancestry (EUR), along the axis towards African ancestry (AFR axis), African ancestry (AFRAFR), and along the axis towards East Asian ancestry (ASN axis) (Figure S2).

### 2.6 Ancestry‐Phenotype Association Analysis

Association between ancestry and categorical phenotypes was evaluated with multinomial or binomial logistic regression. As 13/15 survivors with AFRAFR ancestry received 400 mg/m^2^ of cisplatin (Table S1), associations between genetic ancestry and cisplatin‐induced neurotoxicities were evaluated for the subset of patients receiving 400 to 450 mg/m^2^ of cisplatin to ensure that disparities were not related to higher cisplatin dosage. The patients in this subset received four cycles of cisplatin‐based chemotherapy, with dosage extrapolated by study staff using medical records and body surface area (Table S1). For HL, the Kruskal–Wallis test determined if significant differences existed between geometric means of bilateral hearing thresholds. Multiple pairwise comparisons were conducted with Wilcoxon rank‐sum tests (Benjamini–Hochberg *p*‐value adjustment). The same multiple pairwise comparisons with Wilcoxon rank‐sum tests were conducted to compare age at time of questionnaire between populations (for those receiving four cycles of cisplatin). For clinical and lifestyle factors, which are likely not related to cisplatin dose, as well as self‐reported health, associations between genetic ancestry and phenotype were evaluated across all doses.

### 2.7 Identify SNPs With Population Allele Frequency Differences

Single nucleotide polymorphisms (SNP) genotyped in all survivors after QC were compiled. To filter variants with allele frequency differentiation between populations, *F*_ST_DB, a database with the fixation index (*F*_ST_), a metric for calculating population differentiation, was used. This database calculated the *F*_ST_ for each SNP in 1000 Genomes using the unbiased estimator described by Weir and Cockerham for all population pairings [26]. SNPs were filtered for those with *F*_ST_ > 0.25.

### 2.8 Identify SNPs Impacting Gene Expression and/or Splicing

Genotype‐Tissue Expression Project (GTEx) portal data (V8 release) were used to identify SNPs that were expression quantitative trait loci (eQTL) or splicing quantitative trait loci (sQTL) in tibial nerve or brain tissue [27], excluding those associated only with long non‐coding RNAs (lncRNAs), open reading frames, antisense transcripts, and reverse strands.

SNPs that were eQTLs and/or sQTLs (using GTEx V8 data) for genes involved in cisplatin uptake, metabolism, or effect across tissues were included [28, 29, 30] (Table S2). SNPs were subject to the same *F*_ST_ threshold.

The National Cancer Institute's (NCI) LDmatrix tool was used to calculate the *R* ^2^ between SNP pairs across all 1000 Genomes populations to determine linkage disequilibrium (LD) between SNPs [31]. For SNPs with *R* ^2^ > 0.75, one independent SNP was retained. For cisplatin‐associated SNPs, variants were retained that were not duplicates or in LD with variants kept by nerve/brain eQTL/sQTL filtering (unless the SNP in LD was not an eQTL/sQTL for the cisplatin‐associated gene).

### 2.9 Genotype‐Toxicity Association Analysis

SNPs passing filters were evaluated using logistic regression for association between genotype and phenotype with covariates for age at questionnaire and 10 genetic principal components (PCs). Genetic PCs were included to adjust for population structure (critical due to the absence of sufficiently large populations to independently evaluate genotype–phenotype associations in each population).

For SNPs with *p*‐values of association with toxicity < 1.0 × 10^−4^, ancestral alleles were determined with Ensembl Genome Browser [32].

### 2.10 Gene Expression and Cisplatin Toxicity in Cell Lines

For genes of interest, gene expression in cancer cell lines was obtained from DepMap's Batch corrected Expression Public 24Q2 data [33] (https://depmap.org/portal). Sensitivity to cisplatin was obtained from The Genomics of Drug Sensitivity in Cancer Project [34]. Spearman's rank correlation test between gene expression (log_2_(TPM + 1)) and sensitivity of cancer cell lines to cisplatin (area under the curve (AUC)) (*n* = 575 with nonmissing data) was performed.

### 2.11 Statistical Analysis

Odds ratios (OR) for genetic ancestry group versus categorical/binary phenotypes (PSN, tinnitus, vertigo, and clinical/lifestyle variables) were presented based on logistic regression with 95% confidence intervals and *p*‐values. The exceptions were for tinnitus, diabetes medication use, and current smoking, which had too small a number of cases in some groups that led to unstable OR. Therefore, in these instances, the *p*‐value alone was reported. For hearing loss, the *p*‐value for the Kruskal–Wallis test as well as *p*‐values between individual groups based on multiple pairwise comparisons with the Wilcoxon rank‐sum test (Benjamini–Hochberg adjustment) were reported. OR and *p*‐values were also presented based on logistic regression analyses between SNP genotype and neurotoxicity phenotype for those that passed the suggestively significant *p*‐value threshold (*p* < 1 × 10^−4^), with the significant *p*‐value threshold defined using Bonferroni correction. Spearman's rank correlation coefficients as well as *p*‐values were calculated and reported for the relationship between gene expression and cisplatin sensitivity in cancer cell lines. All analyses were performed in R. 4.1.2.

## 3 Results

**FIGURE 1:**
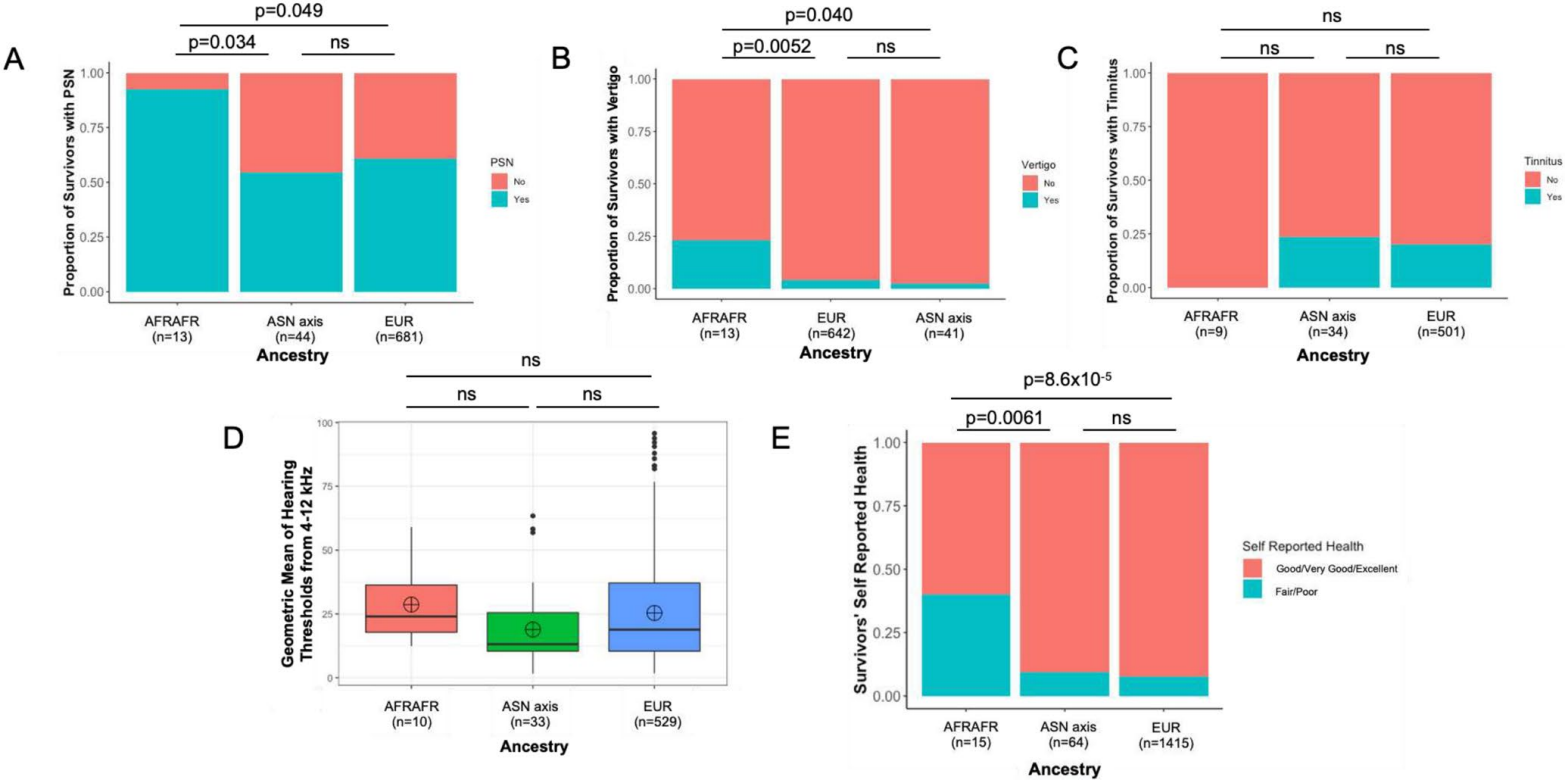
Association between genetic ancestry and toxicity phenotypes. (A) Association between PSN and ancestry for survivors receiving four cycles of cisplatin. (B) Association between vertigo and ancestry for survivors receiving four cycles of cisplatin. (C) Association between tinnitus and ancestry for survivors receiving four cycles of cisplatin. (D) Association between hearing thresholds and ancestry for survivors receiving four cycles of cisplatin. (E) Association between self‐reported health and ancestry for all survivors across cisplatin doses. AFRAFR, African ancestry; ASN axis, Asian axis ancestry; EUR, European ancestry; PSN, Peripheral Sensory Neuropathy.

Using genetic ancestry demarcations (Figure S2), we evaluated each population for the proportion of survivors receiving four cycles of cisplatin with PSN, tinnitus, vertigo, and HL. Survivors with AFRAFR ancestry had a significantly increased prevalence of cisplatin‐induced PSN (Figure 1A) and vertigo (Figure 1B), but not tinnitus (Figure 1C) versus those with EUR ancestry or ASN axis ancestry. As shown in Table 1, for PSN (yes/no), the OR was 10.0 for the AFRAFR vs. ASN axis population (*p* = 3.4 × 10^−2^) and was 7.8 for the AFRAFR vs. EUR population (*p* = 4.9 × 10^−2^). For vertigo, the OR was 12.0 for the AFRAFR vs. ASN axis population (*p* = 4.0 × 10^−2^) and 6.8 for the AFRAFR vs. EUR population (*p* = 5.2 × 10^−3^) (Table 1). The ASN axis population had less HL than the EUR and AFRAFR populations (Figure 1D) yet it was not statistically significant, as multiple pairwise comparisons using the Wilcoxon rank sum test with Benjamini–Hochberg adjustment yielded *p* = 0.072 between AFRAFR and ASN axis survivors and *p* = 0.12 between EUR and ASN axis survivors. The overall Kruskal–Wallis test *p*‐value was 0.09. Since age is associated with cisplatin‐induced neurotoxicities [5], age was assessed between ancestral populations (Table S1). Multiple pairwise comparisons showed that the ASN axis population had a significantly lower age at questionnaire than EUR (*p* = 1.2 × 10^−5^) and AFRAFR (*p* = 0.004) populations, with no significant difference between EUR and AFRAFR populations (*p* = 0.38) among those receiving four cycles of cisplatin (Table S1), perhaps explaining less HL in the ASN axis population as well as less neuropathy and vertigo in this population compared to those of African ancestry. African ancestry survivors were significantly more likely to self‐report health as “fair/poor” versus “good/very good/excellent” compared to other populations, with an OR = 6.4 for AFRAFR vs. ASN axis (*p* = 6.1 × 10^−3^) and an OR = 8.2 for AFRAFR vs. EUR (*p* = 8.6 × 10^−5^) (Figure 1E).

**TABLE 1:** Association between genetic ancestry and cisplatin‐induced neurotoxicities for Platinum Study survivors receiving cisplatin dose 400–450 mg/m^2^.

	ASN axis vs. EUR		AFRAFR vs. ASN axis		AFRAFR vs. EUR	
	OR (95% CI)	*p*	OR (95% CI)	*p*	OR (95% CI)	*p*
PSN (Quite a Bit/Very Much vs. No)	1.2 (0.53, 2.7)	0.67	6.7 (0.61, 73)	0.12	8.0 (0.82, 77)	7.4 × 10^−2^
PSN (Yes vs. No)	0.78 (0.42, 1.5)	0.42	10.0 (1.7, 190)	3.4 × 10^−2^	7.8 (1.5, 142)	4.9 × 10^−2^
Vertigo	0.57 (0.032, 2.8)	0.59	12.0 (1.4, 260)	4.0 × 10^−2^	6.8 (1.5, 24)	5.2 × 10^−3^
Tinnitus	1.2 (0.51, 2.7)	0.62	—	0.99	—	0.99

*Note:* All survivors in subset received 4 cycles of cisplatin chemotherapy, with doses extrapolated from medical records (chemotherapy dose abstract forms) and body surface area (BSA) based on height and weight at the time of chemotherapy. Over 97% of survivors in subset received 400 mg/m^2^ of cisplatin, including all patients with African ancestry.

Abbreviations: AFRAFR, African ancestry; ASN axis, Asian axis ancestry; CI, Confidence Interval; EUR, European ancestry; OR, odds ratio; PSN, peripheral sensory neuropathy.

In efforts to assess whether clinical and/or lifestyle factors contributed to population disparities in cisplatin‐induced neurotoxicities, we evaluated differences in medication use for cholesterol, blood pressure, and diabetes as well as lifestyle factors (smoking and drinking) across populations. Across cisplatin doses, no clinical or lifestyle factors were significantly different between populations, except the ASN axis population had significantly lower anti‐hypertensive medication use versus other populations (ASN axis vs. EUR (OR = 0.11; *p* = 3.2 × 10^−2^); AFRAFR vs. ASN axis (OR = 24; *p* = 6.3 × 10^−3^)) (Table S3), and lower cholesterol medication use vs. EUR (OR = 0.14; *p* = 5.0 × 10^−2^). However, this could perhaps reflect their younger age.

**FIGURE 2:**
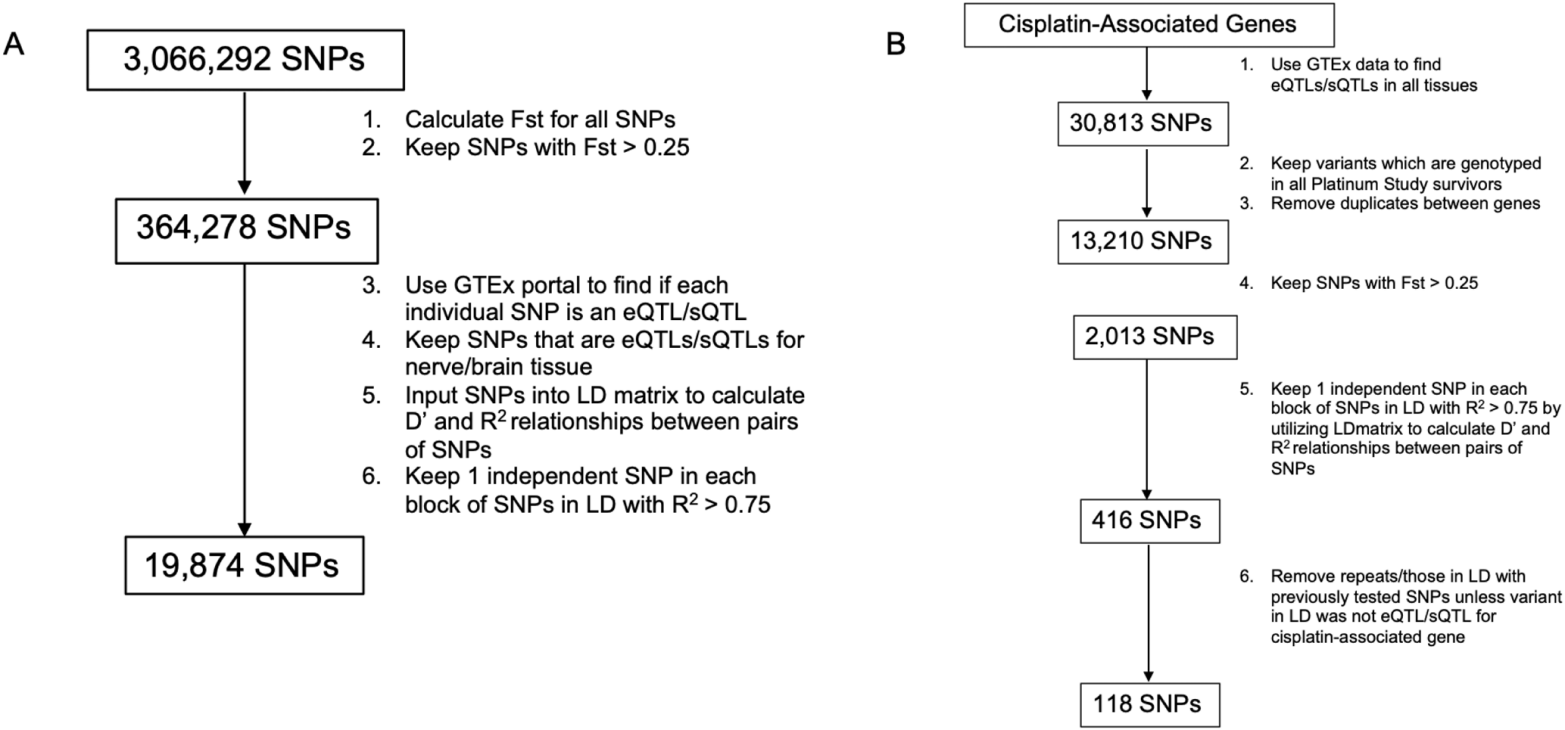
Filtering SNPs for population pharmacogenomic analysis. (A) Genome‐wide approach to select SNPs for genotype–phenotype analysis between independent, functional, ancestry‐informative SNPs and neurotoxicity phenotype. (B) Candidate gene approach to select cisplatin‐associated SNPs for genotype–phenotype analysis between independent, functional, ancestry‐informative, cisplatin‐associated SNPs and neurotoxicity phenotype. *F*_ST_, fixation index; GTEx, Genotype‐Tissue Expression Project; LD, linkage disequilibrium; SNP, Single Nucleotide Polymorphism; eQTL, expression quantitative loci; sQTL, splicing quantitative trait loci.

For the genome‐wide approach, we filtered 3,066,292 SNPs for those with *F*_ST_ > 0.25, leaving 364,278 SNPs. We evaluated for eQTLs/sQTLs in nerve and/or brain tissue, followed by LD determination (*R* ^2^ > 0.75), leaving 19,874 SNPs for analysis (Figure 2A). We also used a candidate gene approach, identifying 30,813 SNPs that were eQTLs/sQTLs for cisplatin‐associated genes across all tissues, of which 13,210 were genotyped (no duplicates). Upon *F*_ST_ filtering, 2013 SNPs remained, with 416 SNPs after LDmatrix filtering. We ensured no duplication or LD with SNPs from the genome‐wide approach, leaving 118 SNPs (Figure 2B). Using both approaches, 19,992 SNPs were included.

We evaluated genotype versus overall PSN prevalence (yes/no; *n* = 1513) and vertigo (*n* = 1427). Although no SNPs passed the Bonferroni threshold (p < 2.5 × 10^−6^), 2 SNPs for PSN and 4 SNPs for vertigo passed a suggestive *p*‐value threshold (p < 1.0 × 10^−4^) (Table 2). In the AFRAFR population, the allele associated with increased PSN for rs2063249 had decreased frequency versus others, while for rs34904346, the allele associated with increased PSN had increased frequency. For vertigo, all 4 SNPs showed increased frequency of the allele associated with increased vertigo in the AFRAFR population versus others. For both SNPs associated with PSN (rs2063249, rs34904346) and 2/4 associated with vertigo (rs28516482, rs2297771), the ancestral allele was the same as the allele most frequent in the AFRAFR population, indicating shifting allele frequencies in the ASN axis and EUR populations. For 2 SNPs associated with vertigo (rs3777909, rs56819906), the ancestral allele was not the allele most frequent in the AFRAFR population, indicating shifting allele frequencies associated with increased cisplatin‐induced vertigo (Table 2). SNPs with *p* < 5.0 × 10^−3^ for PSN and vertigo are in Tables S4 and S5, respectively.

**TABLE 2:** SNPs with association between genotype and phenotype with *p*‐value < 1.0 × 10^−4^.

Neurotoxicity	SNP	chr	*F*_ST_	Genotype	Category Yes vs. No		Allele more frequent in AFRAFR than EUR/ASN axis populations?	Ancestral Allele (Ensembl)	eQTL/sQTL gene	eQTL/sQTL tissue
					OR	*p*				
PSN	rs2063249	5	0.31	CC	Ref.	Ref.	C	C	eQTL—COMMD10	Brain
				CT	1.6	5.3 × 10^−3^				
				TT	2.0	2.8 × 10^−5^				
	rs34904346	20	0.26	AA	Ref.	Ref.	T	T	eQTL—RNF24	Nerve‐tibial
				AT	1.7	1.7 × 10^−4^				
				TT	1.9	2.0 × 10^−5^				
Vertigo	rs28516482	5	0.26	AA	Ref.	Ref.	A	A	sQTL—PRELID	Nerve‐tibial
				AG	0.13	1.4 × 10^−3^				
				GG	0.091	5.0 × 10^−5^				
	rs3777909	6	0.27	AA	Ref.	Ref.	A	G	eQTL—MFSD4B	Nerve‐tibial
				AG	0.14	6.6 × 10^−6^			eQTL—REV3L	Brain
				GG	0.21	3.1 × 10^−5^				
	rs2297771	9	0.38	AA	Ref.	Ref.	A	A	eQTL – ODF2, SPTAN1	Brain
				AG	0.057	6.4 × 10^−6^			sQTL – WDR34, SPTAN1	Nerve‐tibial
				GG	0.098	3.0 × 10^−5^			sQTL – WDR34, CERCAM	Brain
	rs56819906	11	0.34	AA	ref.	ref.	A	G	eQTL‐ DDX25, CDON	Nerve‐tibial
				AG	0.19	1.5 × 10^−3^				
				GG	0.14	7.6 × 10^−5^				

Abbreviations: AFRAFR, African ancestry; ASN axis, Asian axis ancestry; eQTL, expression quantitative trait loci; EUR, European ancestry; OR, odds ratio; PSN, peripheral sensory neuropathy; SNP, single nucleotide polymorphism; sQTL, splicing quantitative trait loci.

**FIGURE 3:**
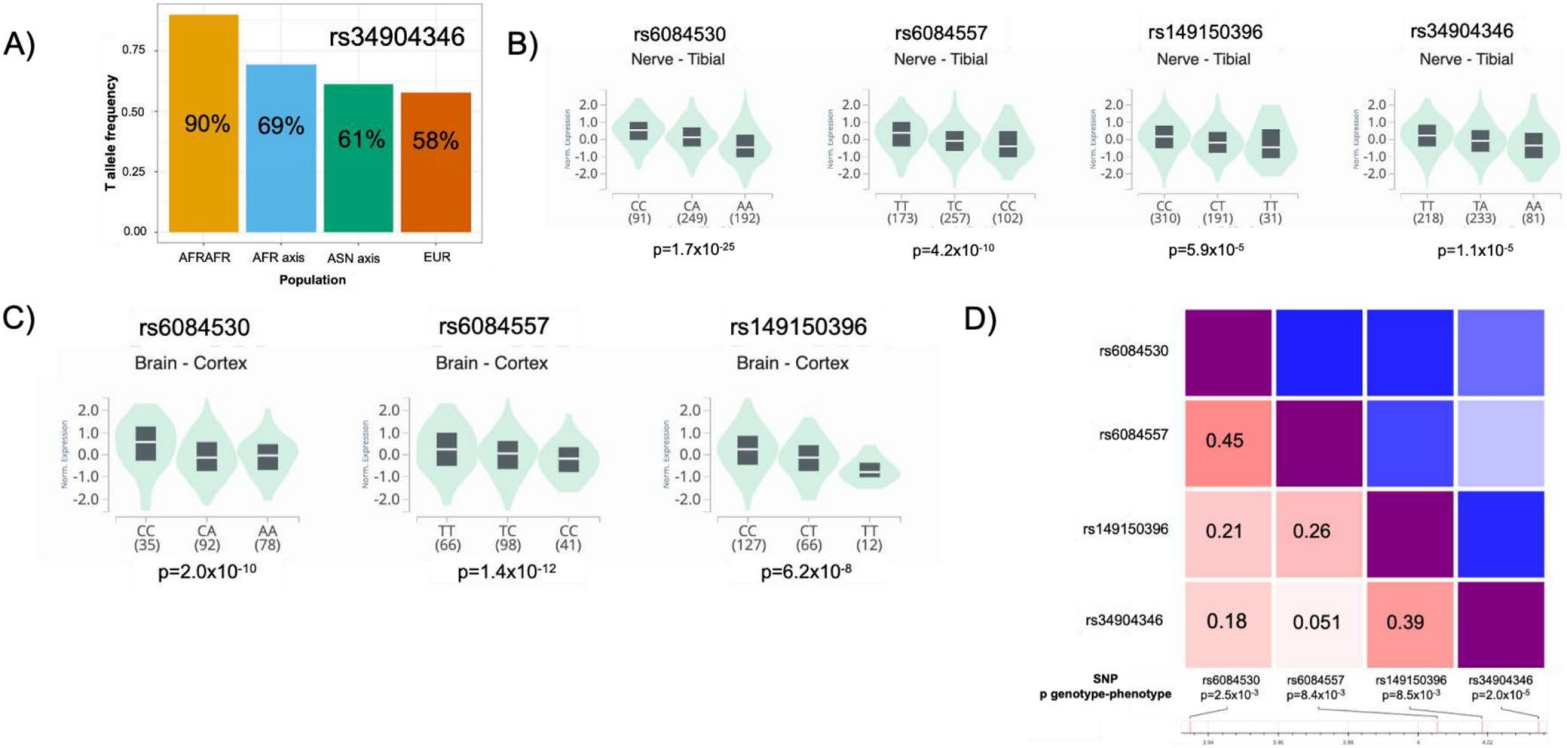
eQTLs for RNF24 are associated with peripheral sensory neuropathy. (A) T allele frequency for rs34904346 in testicular cancer survivors for AFRAFR, AFR axis, ASN axis, and EUR populations. (B) Normalized gene expression and *p*‐value in GTEx for eQTLs for RNF24 in tibial nerve tissue (V8 data). (C) Normalized gene expression and *p*‐value in GTEx for eQTLs for RNF24 in brain cortex tissue (V8 data). (D) Heatmap matrix of pairwise linkage disequilibrium statistics (*R* ^2^ labeled) for eQTLs for RNF24 with association between SNP genotype and PSN phenotype (*p* < 0.01 for all). (B, C) were generated using GTEx V8 data from the GTEx portal between 10/2023 and 12/2023, with visualization created in 05/2024. (D) was adapted from NCI LDmatrix. eQTL, expression quantitative loci; AFRAFR, African ancestry; AFR axis, African axis ancestry; ASN axis, Asian axis ancestry; EUR, European ancestry; GTEx, Genotype‐Tissue Expression Project; SNP, single nucleotide polymorphism.

For PSN (yes vs. no), the associated SNP with the strongest *p*‐value (*p* = 2.0 × 10^−5^) was rs34904346, with an *F*_ST_ = 0.26 (Table 2). In our survivors, the T allele frequency was 90%, 69%, 61%, and 58% in the AFRAFR, AFR axis, ASN axis, and EUR populations, respectively (Figure 3A). Those with the TT genotype had an OR = 1.9 (*p* = 2.0 × 10^−5^) for PSN prevalence compared to AA, demonstrating increased PSN associated with the T allele. This SNP was an eQTL in tibial nerve tissue for the RNF24 gene, with the TT genotype associated with increased gene expression (Figure 3B). Three other SNPs (rs6084530, rs6084557, rs149150396) with *F*_ST_ > 0.25 and which had an association of at least *p* < 0.01 between SNP genotype and PSN prevalence were eQTLs for RNF24 in nerve/brain tissue (Figure 3B,C,D). None of these SNPs were in LD with rs34904346 (Figure 3D). The direction of association between RNF24 expression and cisplatin sensitivity (AUC) in cancer cells (*n* = 575) was as expected (*R* = −0.052), but not significant (*p* = 0.21).

**FIGURE 4:**
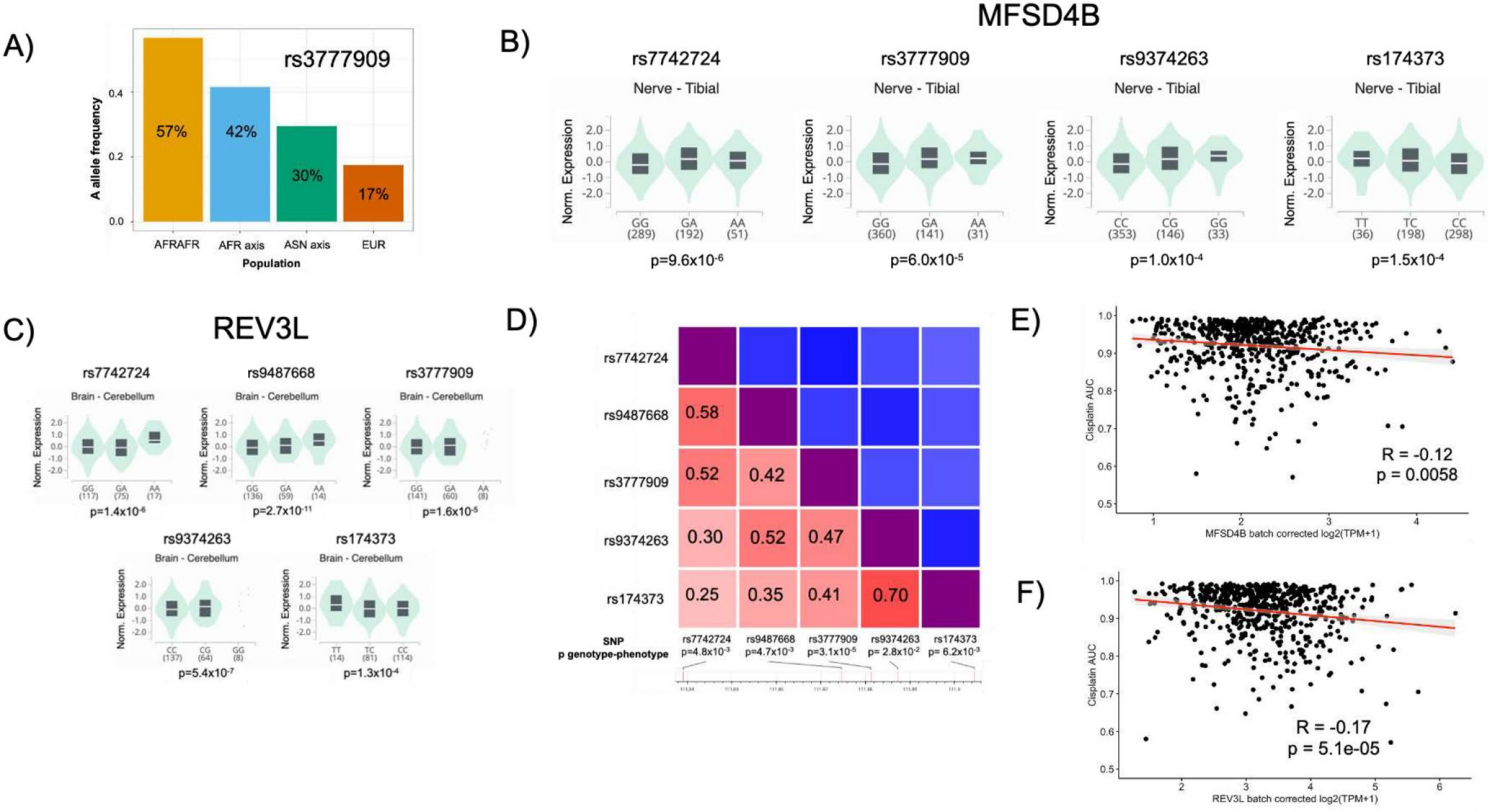
eQTLs for MFSD4B and REV3L are associated with vertigo. (A) A allele frequency for rs3777909 in testicular cancer survivors for AFRAFR, AFR axis, ASN axis, and EUR populations. (B) Normalized gene expression and *p*‐value in GTEx for eQTLs for MFSD4B in tibial nerve tissue (V8 data). (C) Normalized gene expression and *p*‐value in GTEx for eQTLs for REV3L in brain cerebellum tissue (V8 data). (D) Heatmap matrix of pairwise linkage disequilibrium statistics (*R* ^2^ labeled) for eQTLs for MFSD4B/REV3L with association between SNP genotype and vertigo phenotype (*p* < 0.05 for all). (E) Correlation between MFSD4B expression in cancer cell lines and cisplatin AUC. (F) Correlation between REV3L expression in cancer cell lines and cisplatin AUC. (B, C) were generated using GTEx V8 data from the GTEx portal between 10/2023 and 12/2023, with visualization created in 05/2024. (D) was adapted from NCI LDmatrix. AFRAFR, African ancestry; AFR axis, African axis ancestry; ASN axis, Asian axis ancestry; EUR, European ancestry; GTEx, Genotype‐Tissue Expression Project; SNP, single nucleotide polymorphism.

For vertigo, the genotype of SNP rs3777909 was associated with toxicity at *p* = 3.1 × 10^−5^ (*F*_ST_ = 0.27). The A allele had a frequency of 57%, 42%, 30%, and 17% in the AFRAFR, AFR axis, ASN axis, and EUR populations, respectively (Figure 4A). Those with the GG genotype had an OR = 0.21 (*p* = 3.1 × 10^−5^) for vertigo versus AA, indicating increased vertigo risk associated with the A allele (Table 2). This SNP was an eQTL in nerve tissue for MFSD4B (Figure 4B) and in brain tissue for REV3L (Figure 4C). Additional SNPs not in LD with rs3777909 (Figure 4D) that were associated with vertigo at *p* < 0.05 were also eQTLs/sQTLs for MFSD4B and REV3L (Figure 4B,C). For MFSD4B, rs7742724, rs9374263, and rs174373 were also eQTLs in nerve tissue, and these SNPs and rs9487668 were also eQTLs for REV3L in brain tissue. Similarly, rs56819906 and rs73626678 (*R* ^2^ = 0.52) were eQTLs for DDX25 in tibial nerve tissue and associated with vertigo, with *p* = 7.6 × 10^−5^ for GG vs. AA for rs56819906 and *p* = 1.8 × 10^−4^ for TT vs. CC for rs73626678 (Table 2; Figure S3).

Consistent with the association of the A allele for rs3777909 with increased MFSD4B and REV3L expression and increased vertigo, there was an inverse relationship between MFSD4B expression and cisplatin AUC in cancer cell lines in silico (*R* = −0.12; *p* = 5.8 × 10^−3^; Figure 4E) and between REV3L expression and cisplatin AUC (*R* = −0.17; *p* = 5.1 × 10^−5^; Figure 4F) in cancer cell lines in silico, indicating that increased MFSD4B and REV3L expression was significantly associated with increased cellular sensitivity to cisplatin.

## 4 Discussion

This study highlights the importance of elucidating population differences in pharmacogenomics and is the first, to our knowledge, to report disparities in cisplatin‐induced neurotoxicities. Testicular cancer survivors of African ancestry reported poorer self‐reported health, which may be attributed to increased PSN and vertigo compared to those of European ancestry. Measured lifestyle variables were not significantly different between survivors of African ancestry versus those of European ancestry, underscoring the potential importance of genetic variation. These disparities are associated, to some extent, with allele frequency differences in genotypes concomitant with gene expression differences in neuronal genes.

### 4.1 Pharmacogenomic Alleles and Environmental Pressures

For four of six alleles (with suggestively significant *p* < 1.0 × 10^−4^), the ancestral allele was the same as the allele most common in the African ancestry population. Many pharmacogenomic alleles have varied allele frequencies across ancestral populations [35]. Allele frequency differences across populations may reflect adaptations to disparate environments and improved responses to unique selective pressures and chronic exposures. SNPs associated with resistance to heavy metals have been identified [20]. A study found inter‐ethnic differences in allele frequencies in multiple SNPs associated with heavy metal burden between Korean, European, and African populations [36]. Vascular toxicities associated with platinum‐based therapy may have shared risk mechanisms with cardiovascular events related to environmental exposure to heavy metals like arsenic [19]. Allele dynamics respond to environmental changes through directional and stabilizing selection [37, 38]. Disparities in cisplatin‐induced toxicities may reflect the evolution of allele frequencies in SNPs related to metal metabolism/resistance across different geographies due to unique selective pressures.

### 4.2 Diversity in GWAS

Most GWAS have focused on patients of European ancestry [39]. In a review of 28 GWAS of anticancer agents, only 3.7% of subjects were of African ancestry; the majority of studies were ancestrally uniform [40]. Although our cohort was predominantly European, we compared cisplatin‐induced neurotoxicities in diverse populations. The cohort composition reflects testicular cancer epidemiology, which has a 2‐to‐5‐fold higher incidence in non‐Hispanic white males versus other populations [41]. Our findings are important in light of increasing testicular cancer rates in racial/ethnic minorities [42] and the applicability to other cisplatin‐treated cancers.

### 4.3 Ancestral Differences in Chemotherapy‐Induced Toxicities

Studies have demonstrated increased paclitaxel‐induced neuropathy in breast cancer survivors of African versus European ancestry [11, 12, 13, 14]. In a phase III clinical trial, the frequency of Grade 2 to 4 peripheral neuropathy was 43.3% in survivors of African descent versus 24.6% for other patients (HR = 2.1, *p* = 5.6 × 10^−16^) [11]. In another study, survivors of African ancestry (versus European ancestry) had significantly more Grade 3 to 4 taxane‐induced peripheral neuropathy (OR = 2.9, *p* = 2.4 × 10^−11^) and more paclitaxel dose reductions (*p* = 6.6 × 10^−6^) [12].

For survivors of pediatric ALL, European American survivors had more vincristine‐induced peripheral neuropathy than African American survivors (*p* = 0.007) and more neurotoxicity‐related dose reductions (*p* < 0.0001) [15, 16], likely due to vincristine metabolism being impacted by CYP3A5 expression [43]. Functional CYP3A5 enzymes are dependent on the CYP3A5*1 allele, which is more prevalent in those with African versus European ancestry [16].

African American head and neck cancer patients had an increased risk of cisplatin‐induced renal dysfunction (*p* = 0.01) [44] and an increased relative percentage change in eGFR versus White patients (*p* = 0.03) [45].

### 4.4 RNF24, MFSD4B, REV3L in Cisplatin‐Induced Neurotoxicity

Multiple eQTLs for RNF24 were among the top‐associated SNPs for PSN, with one SNP with *p* = 2.0 × 10^−5^ and three other SNPs with *p* < 0.01, placing them among the top 1% of strongest associations tested (out of 19,992 SNPs). RNF24 regulates intracellular trafficking of transient receptor potential cation channel subfamily C proteins (TRPC), and expression at the cell surface [46]. TRPCs are Ca^2+^ permeable channels involved in neurite growth and intracellular Ca^2+^ levels are critical for the balance between neuronal survival and death [47, 48]. Changes in RNF24 gene expression may be associated with changes in TRPC insertion into the membrane, thus disrupting neuronal Ca^2+^ levels, potentially increasing susceptibility to death. Calcium levels are important for cisplatin‐induced injury, as a variant in ACYP2 involved in calcium homeostasis is associated with increased cisplatin‐induced ototoxicity [49] and changes in calcium entry/efflux impact cisplatin‐induced cell death [50, 51]. TRPC levels may also protect against cisplatin‐induced neuronal injury by changing levels of apoptotic proteins [48].

For vertigo, rs3777909 (*p* = 3.14 × 10^−5^) was an eQTL for MFSD4B in nerve and REV3L in brain tissue, with SNPs not in LD also being eQTLs for these genes and associated with vertigo at *p* < 0.05, among the top 5% of SNPs with the strongest association with this toxicity. MFSD4B (SLC60A2) is a sodium/glucose transporter and integral membrane component [52]. Solute carrier transporters play an important role in neurodegenerative disorders by mediating ion and neurotransmitter transport [53] and glucose and glycolysis are important for normal neuronal function [54]. REV3L encodes the catalytic subunit of DNA polymerase zeta and is important in translesion DNA synthesis. In glioma cells, REV3L overexpression facilitates cisplatin chemoresistance [55].

## 5 Strengths and Limitations

This study's strengths include its novelty and conduct in a well‐characterized cohort of survivors undergoing rigorous phenotyping, with cisplatin being the only administered neurotoxic agent. In contrast, head and neck cancer patients who receive cisplatin can also receive other ototoxic therapies such as radiotherapy [56]. Furthermore, age‐related comorbidities are unlikely to confound the results presented here because our cohort of TCS is relatively young (mean age at time of questionnaire in years for those receiving 4 cycles of cisplatin: ASN axis 31.5; EUR 39.5; AFRAFR 42.4). TCS provide a unique opportunity to study disparities in cisplatin‐induced neurotoxicities.

Our Platinum Study cohort allowed us to evaluate population differences in cisplatin‐induced neurotoxicities in contrast to other datasets of TCS with cisplatin‐induced toxicity, which were based in Europe (Denmark, the Netherlands, Norway) and have not been able to examine heterogeneous racial groups [57, 58, 59, 60, 61, 62]. Although we had a low number of non‐European survivors, given testicular cancer epidemiology [41], our investigation represents an important first step. Due to the unequal sample sizes for the ancestry categories, this study is preliminary, and it will be important to validate our findings in larger, more racially diverse cohorts of cisplatin‐treated patients (e.g., those with gynecologic cancers). For genotype–phenotype association analyses, although no SNPs passed Bonferroni threshold, there were some SNPs that were suggestively significant that were eQTLs for RNF24 (neuropathy) and MFSD4B/REV3L (vertigo), with other eQTLs for these genes having a nominally significant association with toxicities. Thus, these are potential genes of interest for which differential expression may be related to the risk of cisplatin‐induced neuropathy or vertigo. In the future, they should be functionally validated. These steps are important to eventually ensure personalized precision medicine [63].

## 6 Comment

Disparities are also influenced by social determinants of health. African American breast cancer survivors face acute survivorship challenges that are exacerbated by disparities in care [64]. We evaluated several clinical factors (blood pressure, cholesterol, and diabetes medication) and lifestyle factors (smoking and drinking) between populations for their contribution to disparities in cisplatin‐induced neurotoxicities. However, we did not identify significant differences between the African ancestry population and European ancestry population for these clinical and lifestyle factors that could explain the observed disparities in PSN and vertigo. There may be other factors that may influence toxicities that are not explicitly part of The Platinum Study. These may include access to care, as well as other social determinants of health. In The Platinum Study, we found strong concordance between the classification of patients by self‐reported race and genetic ancestry in our study population for those with both genetic ancestry and self‐reported race/ethnicity information (excluding those who had no answer/no result and those who answered as identifying only with a racial category not specified on the form). This included 93.3% of those of African ancestry that self‐reported as Black; 97.4% of those of European ancestry that self‐reported as White or White/another racial or ethnic category, and 94.6% of those of Asian axis ancestry that self‐reported as Asian or having Hispanic/Latino ethnicity (to account for the Mexican ancestry in Los Angeles, CA (MXL) population in 1000 Genomes included when defining this category). As such, the social and/or environmental conditions that these patients experience may be similar to those who share their genetic background and may interact with genetic factors to contribute to observed disparities. However, the focus of this paper was on genetic ancestry because race is a social construct. We refrain from attributing causality of disparities to genetics but rather highlight differences in risk allele frequencies between populations. Understanding population pharmacogenomics can mitigate adverse drug events across all future survivors.

## 7 Conclusion

With prior knowledge that disparities between ancestral populations exist in paclitaxel and vincristine‐induced neurotoxicities, we sought to determine if there were disparities in cisplatin‐induced neurotoxicities and if risk allele frequency differences between populations contributed to observed differences. We noted that survivors with African ancestry were significantly more likely to have PSN and vertigo versus survivors of European ancestry, with no significant disparities in several clinical and lifestyle factors. Our analysis of the association between independent, functional variants with allele frequency differences between populations and PSN and vertigo phenotypes found that SNPs that impacted the expression of RNF24 in nerve tissue were among those most strongly associated with PSN, and SNPs that impacted the expression of MFSD4B in nerve tissue and REV3L in brain tissue were among those most strongly associated with vertigo. Consistent with this data, MFSD4B and REV3L expression were significantly associated with cisplatin sensitivity when examining cancer cell lines in silico. Understanding the genetic risk factors for cisplatin‐induced neurotoxicities can allow for monitored dosing, as well as the development of effective treatments that benefit all patients regardless of ancestry and can be expanded to other cancers treated with cisplatin.

## Author Contributions

**Swetha Nakshatri:** conceptualization, investigation, writing – original draft, methodology, validation, formal analysis. **Paul C. Dinh Jr:** investigation, writing – review and editing. **Lawrence H. Einhorn:** investigation, writing – review and editing. **Darren R. Feldman:** investigation, writing – review and editing. **Robert J. Hamilton:** investigation, writing – review and editing. **David J. Vaughn:** investigation, writing – review and editing. **Chunkit Fung:** investigation, writing – review and editing. **Christian Kollmannsberger:** investigation, writing – review and editing. **Robert A. Huddart:** investigation, writing – review and editing. **Lois B. Travis:** investigation, writing – review and editing, funding acquisition, project administration. **Nancy J. Cox:** investigation, writing – review and editing. **M. Eileen Dolan:** investigation, writing – original draft, project administration, supervision, conceptualization.

## Ethics Statement

Study procedures were approved by each institution's Human Subject Review Board and conducted in accordance with the U.S. Common Rule.

## Consent

All participants provided written consent for study participation, access to medical records, and genotyping.

## Conflicts of Interest

L.H.E. owns stock Amgen stock not relevant to the study. D.R.F. serves as a consultant/advisor for BioNTech, Telix Pharmaceuticals, Xencor, Renibus Therapeutics, Exelixis, Debiopharm Group, and receives research funding from Telix Pharmaceuticals, Bristol‐Myers Squibb/Roche, Exelixis, Decibel Therapeutics and has a relationship with UpToDate. None of these relationships are relevant to the study.

## Supporting information


**Figure S1:** Study design for testicular cancer survivors and variant quality control for population pharmacogenomic analysis. (A) Quality control for survivors and population stratification using multidimensional scaling. (B) Quality control and imputation for SNPs. AFR, African (axis); AFRAFR, African ancestry; ASN, Asian (axis); EUR, European; HWE, Hardy–Weinberg Equilibrium; IBD, identity by descent; MAF, minor allele frequency; PUR, Puerto Rican; SD, standard deviation; SNP, Single Nucleotide Polymorphism.
**Figure S2:** Multidimensional scaling analysis to assign genetic ancestry to testicular cancer survivors. Multidimensional scaling scores were calculated for testicular cancer survivors and the 1000 Genomes reference population and were plotted. Overlap between MDS scores was used to classify testicular cancer survivors into genetic ancestral populations based on 1000 Genomes reference. EUR included EUR 1000 Genomes populations (GBR, FIN, CEU, TSI). ASN axis included ASN 1000 Genomes populations (CHB, CHS, JPT) and MXL population and AFR axis included AFR 1000 Genomes populations (YRI, LWK, ASW) and PUR population. AFR axis population was further split into those with just African ancestry (AFRAFR). AFR, African; AFRAFR, African ancestry; AMR, American; ASN, Asian; ASW, African Ancestry in SW USA; CEU, Northern Europeans from Utah; CHB, Han Chinese in Beijing, China; CHS, Han Chinese, South China; EUR, European; FIN, Finnish in Finland; GBR, British from England and Scotland; JPT, Japanese in Tokyo, Japan; LWK, Luhya in Webuye, Kenya; MDS, multidimensional scaling; MXL, Mexican Ancestry in Los Angeles, CA, USA; PUR, Puerto Rican in Puerto Rico; TSI, Tuscans from Italy; YRI, Yoruba in Ibadan, Nigeria.
**Figure S3:** eQTLs for DDX25 in nerve‐tibial tissue are associated with vertigo. (A) A allele frequency for rs56819906 in testicular cancer survivors for AFRAFR, AFR axis, ASN axis, and EUR populations. (B) Heatmap matrix of pairwise linkage disequilibrium statistics (*R* ^2^ labeled) for eQTLs for DDX25 with association between SNP genotype and vertigo phenotype (*p* < 1 × 10^−3^ for all). (C) Normalized gene expression and *p*‐value in GTEx for eQTLs for DDX25 in nerve‐tibial tissue. (B) was adapted from NCI LDmatrix. (C) was generated using GTEX v8 data from the GTEx Portal between 10/2023 and 12/2023, with visualization created in 05/2024.
**Table S1:** Age at questionnaire and cisplatin dose across genetic ancestries for testicular cancer survivors receiving four cycles of cisplatin.
**Table S2:** Cisplatin‐associated genes for candidate gene approach.
**Table S3:** Association between genetic ancestry and medication use and lifestyle behaviors for testicular cancer survivors.
**Table S4:** List of SNPs with greatest association between genotype and PSN Yes vs. no phenotype (*p*‐value < 5 × 10^−3^).
**Table S5:** List of SNPs with greatest association between genotype and vertigo phenotype (*p*‐value < 5 × 10^−3^).

## Data Availability

In addition to genotype–phenotype association analysis results in Supplemental Tables S4 and S5, full summary statistics for genotype–phenotype association analysis for cisplatin‐induced peripheral sensory neuropathy and vertigo will be available at *The latest research findings from The Platinum Study* (https://cancer.iu.edu/patients/surviving/platinum‐study/research‐updates.html).
