# Supplementary material for: Impact of Population Pharmacogenomics on Cisplatin‐Induced Neurotoxicities in Testicular Cancer Survivors
Source: Cancer Med. 2025 Sep 5;14(17):e71218. doi: 10.1002/cam4.71218 (PMC12413486; doi:10.1002/cam4.71218)
Supplement: Supplementary file 1 — Figure S1: Study design for testicular cancer survivors and variant quality control for population pharmacogenomic analysis. (A) Quality control for survivors and population stratification using multidimensional scaling. (B) Quality control and imputation for SNPs. AFR, African (axis); AFRAFR, African ancestry; ASN, Asian (axis); EUR, European; HWE, Hardy–Weinberg Equilibrium; IBD, identity by descent; MAF, minor allele frequency; PUR, Puerto Rican; SD, standard deviation; SNP, Single Nucleotide Polymorphism. Figure S2: Multidimensional scaling analysis to assign genetic ancestry to testicular cancer survivors. Multidimensional scaling scores were calculated for testicular cancer survivors and the 1000 Genomes reference population and were plotted. Overlap between MDS scores was used to classify testicular cancer survivors into genetic ancestral populations based on 1000 Genomes reference. EUR included EUR 1000 Genomes populations (GBR, FIN, CEU, TSI). ASN axis included ASN 1000 Genomes populations (CHB, CHS, JPT) and MXL population and AFR axis included AFR 1000 Genomes populations (YRI, LWK, ASW) and PUR population. AFR axis population was further split into those with just African ancestry (AFRAFR). AFR, African; AFRAFR, African ancestry; AMR, American; ASN, Asian; ASW, African Ancestry in SW USA; CEU, Northern Europeans from Utah; CHB, Han Chinese in Beijing, China; CHS, Han Chinese, South China; EUR, European; FIN, Finnish in Finland; GBR, British from England and Scotland; JPT, Japanese in Tokyo, Japan; LWK, Luhya in Webuye, Kenya; MDS, multidimensional scaling; MXL, Mexican Ancestry in Los Angeles, CA, USA; PUR, Puerto Rican in Puerto Rico; TSI, Tuscans from Italy; YRI, Yoruba in Ibadan, Nigeria. Figure S3: eQTLs for DDX25 in nerve‐tibial tissue are associated with vertigo. (A) A allele frequency for rs56819906 in testicular cancer survivors for AFRAFR, AFR axis, ASN axis, and EUR populations. (B) Heatmap matrix of pairwise linkage disequilibrium statistics ( [file CAM4-14-e71218-s001.docx]

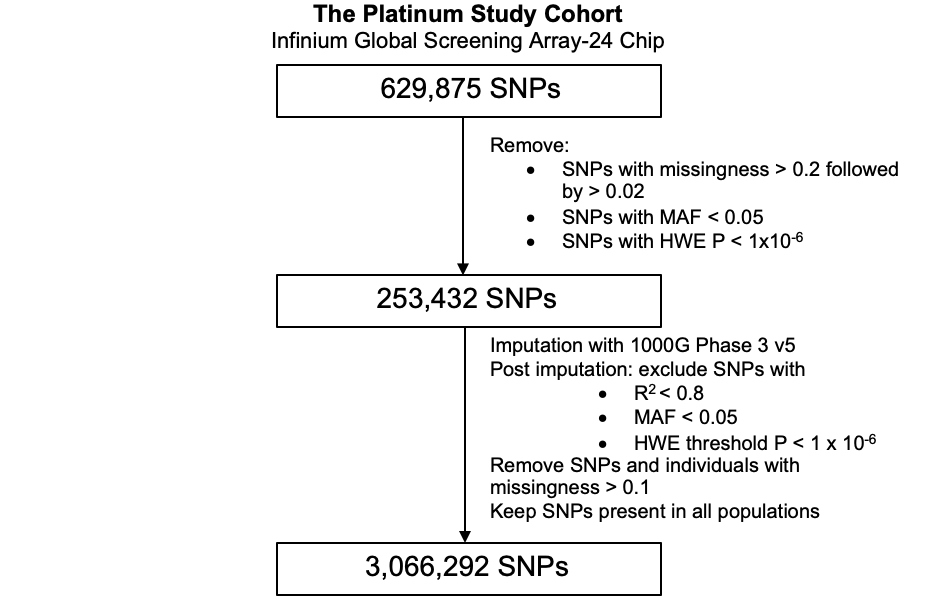


**Supplemental Figure 1. Study Design for Testicular Cancer Survivors and Variant Quality Control for Population Pharmacogenomic Analysis. A,** Quality control for survivors and population stratification using multidimensional scaling. **B,** Quality control and imputation for SNPs. SNP = Single Nucleotide Polymorphism, MAF = minor allele frequency, HWE = Hardy-Weinberg Equilibrium, IBD = identity by descent, SD = standard deviation, EUR = European, AFR = African (axis), AFRAFR = African ancestry, PUR = Puerto Rican, ASN = Asian (axis).


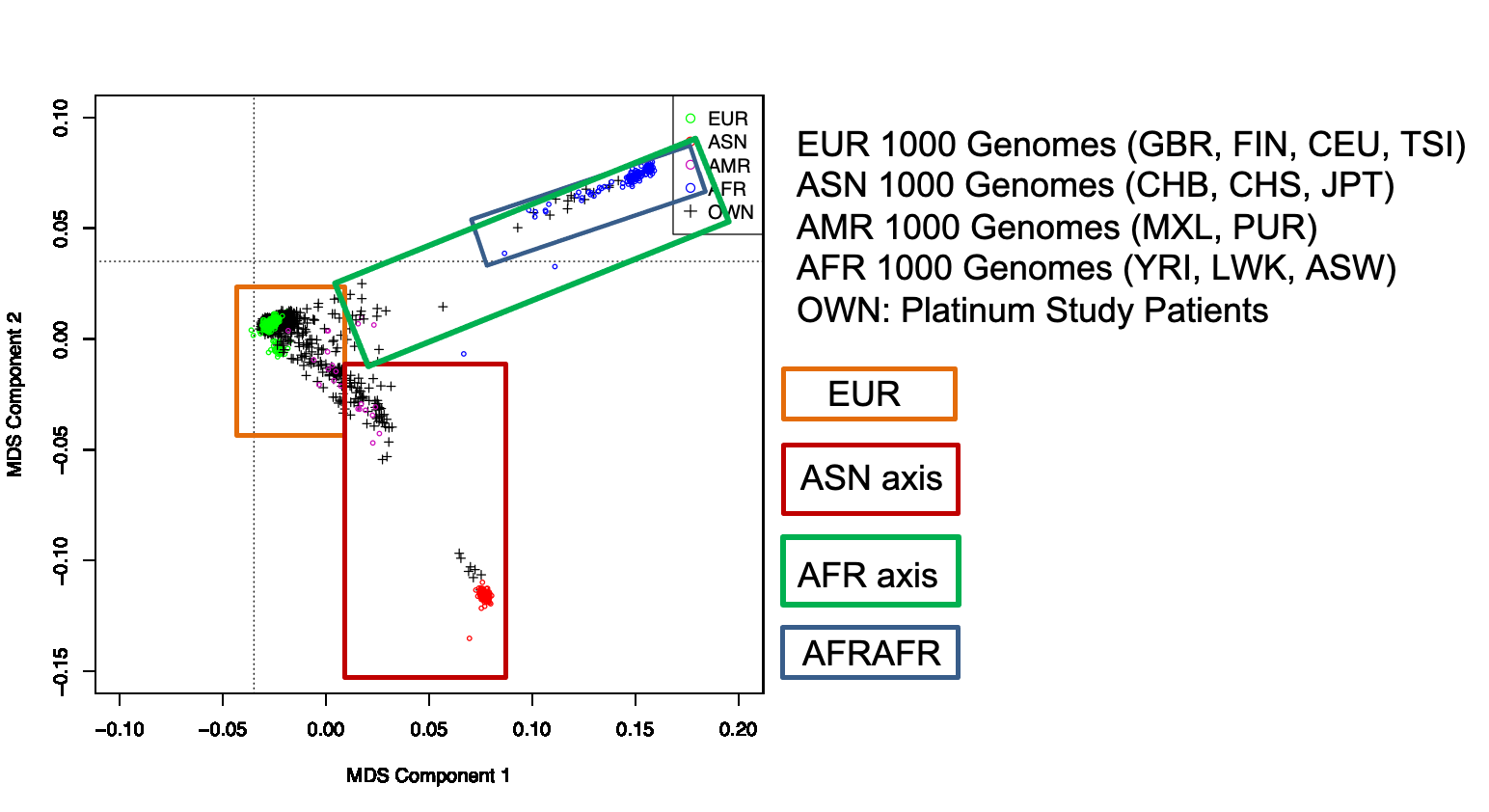


**Supplemental Figure 2. Multidimensional Scaling Analysis to Assign Genetic Ancestry to Testicular Cancer Survivors.** Multidimensional scaling scores were calculated for testicular cancer survivors and the 1000 Genomes reference population and were plotted. Overlap between MDS scores was used to classify testicular cancer survivors into genetic ancestral populations based on 1000 Genomes reference. EUR included EUR 1000 Genomes populations (GBR, FIN, CEU, TSI). ASN axis included ASN 1000 Genomes populations (CHB, CHS, JPT) and MXL population and AFR axis included AFR 1000 Genomes populations (YRI, LWK, ASW) and PUR population. AFR axis population was further split into those with just African ancestry (AFRAFR). MDS = multidimensional scaling, EUR = European, AFR = African, ASN = Asian, AFRAFR = African ancestry, AMR = American, GBR = British from England and Scotland, FIN = Finnish in Finland, CEU = Northern Europeans from Utah, TSI = Tuscans from Italy, CHB = Han Chinese in Beijing, China, CHS = Han Chinese, South China, JPT = Japanese in Tokyo, Japan, MXL = Mexican Ancestry in Los Angeles, CA, USA, PUR = Puerto Rican in Puerto Rico, YRI = Yoruba in Ibadan, Nigeria, LWK = Luhya in Webuye, Kenya, ASW = African Ancestry in SW USA.

**Supplemental Table 1. Age at Questionnaire and Cisplatin Dose across Genetic Ancestries for Testicular Cancer Survivors Receiving Four Cycles of Cisplatin**

| Characteristic | AFRAFR (n=13) | EUR (n=701) | ASN axis (n=45) |
| --- | --- | --- | --- |
| Cisplatin Dose (mg/m^2^) | | | |
| Mean | 400 | 400.36 | 400.20 |
| 400 | 13 | 684 | 44 |
| >400-410 | 0 | 8 | 1 |
| $\geq$410-420 | 0 | 2 | 0 |
| $\geq$420-430 | 0 | 5 | 0 |
| $\geq$430-450 | 0 | 2 | 0 |
| Age at Questionnaire (years) | | | |
| Mean | 42.4 | 39.5 | 31.5 |
| <20 | 0 | 5 | 1 |
| $\geq$20-30 | 2 | 157 | 18 |
| $\geq$30-40 | 4 | 229 | 23 |
| $\geq$40 | 7 | 300 | 3 |
| Not available | 0 | 10 | 0 |

Abbreviations: ASN, Asian axis ancestry; EUR, European ancestry; AFRAFR, African ancestry.

**Supplemental Table 2. Cisplatin-Associated Genes for Candidate Gene Approach.**

| Category | Gene |
| --- | --- |
| Antioxidant Protection | GSTM1, GSTP1, GSTO1 |
| Import (main) | SLC31A1 (CTR1), SLC7A2 (CTR2) |
| Export | ATP7B, ABCC2 |
| DNA repair | 1. XPA, ERCC1/2/4 2. POLQ, POLN, POLD1, PNKP, BARD1, PARP1 3. FEN1, FAAP24 |
| Metallothionine | MT1A, MT2A, MT3, MT4, MTR1, MTF-1, ZBTB1, USF1, SPI1 (PU.1), SMARCA4 (BRG1), MBD3, CEBP$\alpha$, XRCC5 (KU80), NFIA (NF1), DNMT3A |
| Other SLC transporters | SLC22A5/7, SLC22A10-13, SLC2216, SLC22A20, SLC31A2 |
| GSH synthesis | GSS, GCLC, GCLM |
| Apoptosis | 1. BCL2, BCL2L1, TP63 2. CFLAR |
| Protein Kinase | PRKCA, PRKCB |
| Mitogen-active protein kinase | MAPK3,MAPK13,MAP3K1 |
| Transcription factors | NFKB1/2, TWIST, SNAI1 |
| Miscellaneous | FOS, CDKN2C, TLR4 |

Abbreviations: SLC, solute carrier; GSH, glutathione.

**Supplemental Table 3. Association between Genetic Ancestry and Medication Use and Lifestyle Behaviors for Testicular Cancer Survivors.**

|  | ASN axis vs. EUR | | AFRAFR vs. ASN axis | | AFRAFR vs. EUR | |
| --- | --- | --- | --- | --- | --- | --- |
|  | OR (95% CI) | p-value | OR (95% CI) | p-value | OR (95% CI) | p-value |
| Cholesterol Medication (Y vs. N) | 0.14 (0.0078, 0.63) | 5.0x10^-2^ | 9.8 (0.88, 220) | 7.1x10^-2^ | 1.4 (0.21, 5.0) | 0.69 |
| Blood Pressure Medication (Y vs. N) | 0.11 (0.0065, 0.52) | 3.2x10^-2^ | 24 (3.22, 505) | 6.3x10^-3^ | 2.8 (0.76, 8.5) | 8.5x10^-2^ |
| Diabetes Requiring Insulin (Y vs. N) | - | 0.99 | - | 1 | - | 0.99 |
| Diabetes Requiring Tablets/Pills (Y vs. N) | 0.65 (0.036, 3.1) | 0.67 | - | 1.0 | - | 0.98 |
| Ever Smoked (Y vs. N) | 1.4 (0.85, 2.3) | 0.18 | 0.50 (0.14, 1.6) | 0.25 | 0.70 (0.22, 2.0) | 0.52 |
| Current Smoker (Y vs. N) | 0.66 (0.20, 1.6) | 0.42 | - | 1.0 | - | 0.98 |
| Excess Drinking (Y vs. N) | 0.33 (0.079, 0.89) | 6.0x10^-2^ | 5.1 (0.85, 31) | 6.3x10^-2^ | 1.7 (0.37, 5.3) | 0.44 |

Abbreviations: ASN, Asian axis ancestry; EUR, European ancestry; AFRAFR, African ancestry; OR, odds ratio; CI, confidence interval; Y, Yes; N, No.

**Supplemental Table 4.** **List of SNPs with Greatest Association between Genotype and PSN Yes vs. No Phenotype (p-value < 5x10^-3^)**

| SNP | chromosome | F_ST_ | Genotype | PSN Category Yes vs. No | |
| --- | --- | --- | --- | --- | --- |
|  |  |  |  | OR | p-value |
| rs34904346 | 20 | 0.26 | AA | ref. | ref. |
|  |  |  | AT | 1.7 | 1.7x10^-4^ |
|  |  |  | TT | 1.9 | 2.0x10^-5^ |
| rs2063249 | 5 | 0.32 | CC | ref. | ref. |
|  |  |  | CT | 1.6 | 5.3x10^-3^ |
|  |  |  | TT | 2.0 | 2.8x10^-5^ |
| rs1778483 | 6 | 0.47 | GG | ref. | ref. |
|  |  |  | GT | 1.8 | 1.6x10^-3^ |
|  |  |  | TT | 2.1 | 1.2x10^-4^ |
| rs4236999 | 8 | 0.30 | CC | ref. | ref. |
|  |  |  | CT | 1.2 | 0.23 |
|  |  |  | TT | 1.9 | 2.7x10^-4^ |
| rs9494195 | 6 | 0.39 | AA | ref. | ref. |
|  |  |  | AG | 0.89 | 0.30 |
|  |  |  | GG | 2.5 | 3.1x10^-4^ |
| rs1150721 | 6 | 0.59 | CC | ref. | ref. |
|  |  |  | CG | 1.6 | 4.1x10^-3^ |
|  |  |  | GG | 1.8 | 3.9x10^-4^ |
| rs10213186 | 4 | 0.26 | AA | ref. | ref. |
|  |  |  | AT | 0.92 | 0.46 |
|  |  |  | TT | 2.3 | 4.2x10^-4^ |
| rs10085965 | 8 | 0.42 | CC | ref. | ref. |
|  |  |  | CT | 0.90 | 0.41 |
|  |  |  | TT | 0.59 | 5.2x10^-4^ |
| rs34015011 | 8 | 0.38 | GG | ref. | ref. |
|  |  |  | GT | 0.79 | 4.0x10^-2^ |
|  |  |  | TT | 0.55 | 7.9x10^-4^ |
| rs8182578 | 19 | 0.32 | AA | ref. | ref. |
|  |  |  | AG | 0.71 | 3.0x10^-2^ |
|  |  |  | GG | 0.58 | 8.3x10^-4^ |
| rs8059496 | 16 | 0.27 | AA | ref. | ref. |
|  |  |  | AG | 2.6 | 4.9x10^-4^ |
|  |  |  | GG | 2.4 | 8.3x10^-4^ |
| rs28481421 | 8 | 0.26 | CC | ref. | ref. |
|  |  |  | CT | 1.5 | 5.1x10^-3^ |
|  |  |  | TT | 1.7 | 8.7x10^-4^ |
| rs3777909 | 6 | 0.27 | AA | ref. | ref. |
|  |  |  | AG | 0.35 | 9.4x10^-4^ |
|  |  |  | GG | 0.36 | 9.0x10^-4^ |
| rs4736347 | 8 | 0.31 | CC | ref. | ref. |
|  |  |  | CT | 1.4 | 1.5x10^-2^ |
|  |  |  | TT | 1.7 | 9.8x10^-4^ |
| rs11024606 | 11 | 0.44 | AA | ref. | ref. |
|  |  |  | AC | 1.1 | 0.70 |
|  |  |  | CC | 1.7 | 1.0x10^-3^ |
| rs8131065 | 21 | 0.36 | CC | ref. | ref. |
|  |  |  | CT | 1.1 | 0.33 |
|  |  |  | TT | 6.1 | 1.0x10^-3^ |
| rs10957641 | 8 | 0.33 | GG | ref. | ref. |
|  |  |  | GT | 1.1 | 0.25 |
|  |  |  | TT | 1.7 | 1.1x10^-3^ |
| rs442439 | 6 | 0.54 | AA | ref. | ref. |
|  |  |  | AG | 1.8 | 1.2x10^-3^ |
|  |  |  | GG | 1.8 | 1.3x10^-3^ |
| rs4488948 | 4 | 0.54 | CC | ref. | ref. |
|  |  |  | CT | 0.83 | 0.21 |
|  |  |  | TT | 0.61 | 1.4x10^-3^ |
| rs4076424 | 8 | 0.41 | CC | ref. | ref. |
|  |  |  | CT | 0.39 | 2.4x10^-3^ |
|  |  |  | TT | 0.38 | 1.4x10^-3^ |
| rs4902768 | 14 | 0.28 | CC | ref. | ref. |
|  |  |  | CT | 1.2 | 0.10 |
|  |  |  | TT | 1.7 | 1.4x10^-3^ |
| rs7243210 | 18 | 0.26 | CC | ref. | ref. |
|  |  |  | CT | 1.4 | 2.4x10^-3^ |
|  |  |  | TT | 1.7 | 1.5x10^-3^ |
| rs2023509 | 20 | 0.36 | CC | ref. | ref. |
|  |  |  | CT | 0.82 | 0.13 |
|  |  |  | TT | 0.61 | 1.57x10^-3^ |
| rs4682783 | 3 | 0.40 | AA | ref. | ref. |
|  |  |  | AG | 0.83 | 0.13 |
|  |  |  | GG | 0.61 | 1.6x10^-3^ |
| rs7136124 | 12 | 0.34 | AA | ref. | ref. |
|  |  |  | AG | 0.78 | 5.3x10^-2^ |
|  |  |  | GG | 0.61 | 1.6x10^-3^ |
| rs900641 | 5 | 0.48 | GG | ref. | ref. |
|  |  |  | GT | 3.9 | 3.5x10^-3^ |
|  |  |  | TT | 4.2 | 1.7x10^-3^ |
| rs532543 | 9 | 0.29 | AA | ref. | ref. |
|  |  |  | AG | 0.89 | 0.32 |
|  |  |  | GG | 0.38 | 1.8x10^-3^ |
| rs11874776 | 18 | 0.57 | CC | ref. | ref. |
|  |  |  | CT | 1.2 | 0.29 |
|  |  |  | TT | 0.18 | 1.8x10^-3^ |
| rs7694450 | 4 | 0.39 | AA | ref. | ref. |
|  |  |  | AG | 1.4 | 1.0x10^-2^ |
|  |  |  | GG | 1.6 | 1.8x10^-3^ |
| rs28459852 | 8 | 0.37 | AA | ref. | ref. |
|  |  |  | AT | 1.4 | 2.0x10^-2^ |
|  |  |  | TT | 1.6 | 1.8x10^-3^ |
| rs303860 | 3 | 0.38 | AA | ref. | ref. |
|  |  |  | AG | 1.2 | 0.13 |
|  |  |  | GG | 0.42 | 1.9x10^-3^ |
| rs213228 | 6 | 0.29 | AA | ref. | ref. |
|  |  |  | AC | 0.80 | 5.1x10^-2^ |
|  |  |  | CC | 0.57 | 2.0x10^-3^ |
| rs4833705 | 4 | 0.40 | CC | ref. | ref. |
|  |  |  | CT | 0.90 | 0.35 |
|  |  |  | TT | 0.47 | 2.0x10^-3^ |
| rs11650955 | 17 | 0.27 | AA | ref. | ref. |
|  |  |  | AG | 1.3 | 2.8x10^-2^ |
|  |  |  | GG | 1.6 | 2.1x10^-3^ |
| rs3103583 | 5 | 0.38 | CC | ref. | ref. |
|  |  |  | CT | 0.86 | 0.27 |
|  |  |  | TT | 0.28 | 2.1x10^-3^ |
| rs558811 | 2 | 0.27 | CC | ref. | ref. |
|  |  |  | CG | 1.1 | 0.52 |
|  |  |  | GG | 1.7 | 2.1x10^-3^ |
| rs4683114 | 3 | 0.40 | CC | ref. | ref. |
|  |  |  | CT | 1.3 | 4.4x10^-2^ |
|  |  |  | TT | 1.6 | 2.1x10^-3^ |
| rs7897357 | 10 | 0.47 | CC | ref. | ref. |
|  |  |  | CT | 1.3 | 4.0x10^-2^ |
|  |  |  | TT | 1.7 | 2.2x10^-3^ |
| rs6717311 | 2 | 0.28 | CC | ref. | ref. |
|  |  |  | CT | 0.88 | 0.28 |
|  |  |  | TT | 0.59 | 2.2x10^-3^ |
| rs12611162 | 19 | 0.25 | AA | ref. | ref. |
|  |  |  | AG | 1.7 | 7.9x10^-3^ |
|  |  |  | GG | 1.8 | 2.2x10^-3^ |
| rs1592457 | 16 | 0.25 | CC | ref. | ref. |
|  |  |  | CT | 1.0 | 0.87 |
|  |  |  | TT | 0.55 | 2.3x10^-3^ |
| rs2869358 | 4 | 0.34 | AA | ref. | ref. |
|  |  |  | AG | 1.1 | 0.59 |
|  |  |  | GG | 1.7 | 2.3x10^-3^ |
| rs249834 | 12 | 0.32 | CC | ref. | ref. |
|  |  |  | CT | 0.70 | 3.4x10^-2^ |
|  |  |  | TT | 0.59 | 2.3x10^-3^ |
| rs9374263 | 6 | 0.28 | CC | ref. | ref. |
|  |  |  | CG | 1.2 | 0.20 |
|  |  |  | GG | 2.7 | 2.3x10^-3^ |
| rs10000663 | 4 | 0.42 | CC | ref. | ref. |
|  |  |  | CT | 0.71 | 2.0x10^-2^ |
|  |  |  | TT | 0.62 | 2.3x10^-3^ |
| rs10766514 | 11 | 0.32 | AA | ref. | ref. |
|  |  |  | AG | 0.46 | 1.2x10^-3^ |
|  |  |  | GG | 0.48 | 2.3x10^-3^ |
| rs34366795 | 3 | 0.33 | AA | ref. | ref. |
|  |  |  | AG | 0.63 | 3.3x10^-3^ |
|  |  |  | GG | 0.61 | 2.4x10^-3^ |
| rs2564117 | 2 | 0.31 | AA | ref. | ref. |
|  |  |  | AT | 1.3 | 6.8x10^-2^ |
|  |  |  | TT | 1.6 | 2.4x10^-3^ |
| rs12674115 | 7 | 0.31 | CC | ref. | ref. |
|  |  |  | CG | 2.5 | 2.6x10^-4^ |
|  |  |  | GG | 2.1 | 2.5x10^-3^ |
| rs6084530 | 20 | 0.26 | AA | ref. | ref. |
|  |  |  | AC | 1.3 | 5.2x10^-2^ |
|  |  |  | CC | 1.7 | 2.5x10^-3^ |
| rs726104 | 21 | 0.34 | AA | ref. | ref. |
|  |  |  | AG | 1.3 | 9.5x10^-2^ |
|  |  |  | GG | 1.6 | 2.5x10^-3^ |
| rs1582958 | 5 | 0.36 | AA | ref. | ref. |
|  |  |  | AG | 0.57 | 1.5x10^-2^ |
|  |  |  | GG | 0.50 | 2.7x10^-3^ |
| rs4739079 | 8 | 0.43 | AA | ref. | ref. |
|  |  |  | AT | 0.99 | 0.93 |
|  |  |  | TT | 0.63 | 2.7x10^-3^ |
| rs11644601 | 16 | 0.26 | CC | ref. | ref. |
|  |  |  | CT | 1.5 | 4.5x10^-2^ |
|  |  |  | TT | 1.8 | 2.8x10^-3^ |
| rs6769051 | 3 | 0.49 | GG | ref. | ref. |
|  |  |  | GT | 1.1 | 0.47 |
|  |  |  | TT | 1.7 | 2.9x10^-3^ |
| rs7809654 | 7 | 0.47 | AA | ref. | ref. |
|  |  |  | AG | 2.4 | 2.3x10^-3^ |
|  |  |  | GG | 2.3 | 3.0x10^-3^ |
| rs8104076 | 19 | 0.34 | AA | ref. | ref. |
|  |  |  | AG | 1.2 | 0.11 |
|  |  |  | GG | 2.4 | 3.0x10^-3^ |
| rs11719526 | 3 | 0.41 | CC | ref. | ref. |
|  |  |  | CT | 1.0 | 0.87 |
|  |  |  | TT | 1.6 | 3.0x10^-3^ |
| rs58018557 | 15 | 0.35 | CC | ref. | ref. |
|  |  |  | CT | 2.1 | 7.6x10^-3^ |
|  |  |  | TT | 2.3 | 3.1x10^-3^ |
| rs7529452 | 1 | 0.27 | CC | ref. | ref. |
|  |  |  | CT | 0.81 | 6.1x10^-2^ |
|  |  |  | TT | 0.57 | 3.1x10^-3^ |
| rs1514967 | 1 | 0.30 | AA | ref. | ref. |
|  |  |  | AC | 1.3 | 3.2x10^-2^ |
|  |  |  | CC | 1.6 | 3.1x10^-3^ |
| rs35558975 | 8 | 0.39 | CC | ref. | ref. |
|  |  |  | CG | 0.93 | 0.59 |
|  |  |  | GG | 0.58 | 3.1x10^-3^ |
| rs156737 | 6 | 0.56 | AA | ref. | ref. |
|  |  |  | AG | 0.87 | 0.25 |
|  |  |  | GG | 0.60 | 3.3x10^-3^ |
| rs11783227 | 8 | 0.31 | GG | ref. | ref. |
|  |  |  | GT | 1.5 | 1.1x10^-2^ |
|  |  |  | TT | 1.8 | 3.4x10^-3^ |
| rs2538046 | 7 | 0.29 | AA | ref. | ref. |
|  |  |  | AG | 0.84 | 0.22 |
|  |  |  | GG | 0.63 | 3.4x10^-3^ |
| rs61812714 | 1 | 0.31 | AA | ref. | ref. |
|  |  |  | AC | 1.6 | 1.5x10^-2^ |
|  |  |  | CC | 1.7 | 3.5x10^-3^ |
| rs1320893 | 7 | 0.30 | AA | ref. | ref. |
|  |  |  | AG | 1.0 | 0.98 |
|  |  |  | GG | 0.59 | 3.5x10^-3^ |
| rs3771605 | 2 | 0.37 | AA | ref. | ref. |
|  |  |  | AG | 1.5 | 6.1x10^-3^ |
|  |  |  | GG | 1.6 | 3.6x10^-3^ |
| rs5750911 | 22 | 0.33 | AA | ref. | ref. |
|  |  |  | AG | 1.0 | 0.89 |
|  |  |  | GG | 0.62 | 3.6x10^-3^ |
| rs6942249 | 6 (cisplatin-associated) | 0.39 | AA | ref. | ref. |
|  |  |  | AG | 1.7 | 2.6x10^-2^ |
|  |  |  | GG | 2.0 | 3.7x10^-3^ |
| rs1119229 | 19 | 0.30 | CC | ref. | ref. |
|  |  |  | CT | 0.55 | 2.4x10^-2^ |
|  |  |  | TT | 0.47 | 3.8x10^-3^ |
| rs11114340 | 12 | 0.30 | CC | ref. | ref. |
|  |  |  | CT | 0.87 | 0.34 |
|  |  |  | TT | 0.63 | 3.9x10^-3^ |
| rs174373 | 6 | 0.29 | CC | ref. | ref. |
|  |  |  | CT | 0.96 | 0.75 |
|  |  |  | TT | 2.3 | 3.9x10^-3^ |
| rs2422391 | 2 | 0.32 | AA | ref. | ref. |
|  |  |  | AG | 1.3 | 5.2x10^-2^ |
|  |  |  | GG | 2.0 | 4.3x10^-3^ |
| rs17423809 | 2 | 0.32 | CC | ref. | ref. |
|  |  |  | CT | 1.5 | 2.2x10^-2^ |
|  |  |  | TT | 1.7 | 4.5x10^-3^ |
| rs56039135 | 2 | 0.28 | AA | ref. | ref. |
|  |  |  | AC | 1.1 | 0.37 |
|  |  |  | CC | 0.42 | 4.5x10^-3^ |
| rs7798564 | 7 | 0.33 | CC | ref. | ref. |
|  |  |  | CT | 2.1 | 3.3x10^-3^ |
|  |  |  | TT | 2.1 | 4.6x10^-3^ |
| rs162556 | 2 | 0.43 | AA | ref. | ref. |
|  |  |  | AG | 0.93 | 0.55 |
|  |  |  | GG | 0.64 | 4.6x10^-3^ |
| rs12542796 | 8 | 0.30 | CC | ref. | ref. |
|  |  |  | CT | 1.5 | 2.1x10^-2^ |
|  |  |  | TT | 1.6 | 4.7x10^-3^ |
| rs4522527 | 19 | 0.32 | AA | ref. | ref. |
|  |  |  | AG | 0.59 | 3.3x10^-3^ |
|  |  |  | GG | 0.60 | 4.7x10^-3^ |
| rs11746505 | 5 | 0.31 | CC | ref. | ref. |
|  |  |  | CT | 1.2 | 0.11 |
|  |  |  | TT | 1.6 | 4.8x10^-3^ |
| rs653407 | 18 | 0.25 | CC | ref. | ref. |
|  |  |  | CT | 1.5 | 3.1x10^-2^ |
|  |  |  | TT | 1.7 | 4.8x10^-3^ |
| rs11024622 | 11 | 0.37 | CC | ref. | ref. |
|  |  |  | CG | 0.70 | 1.2x10^-2^ |
|  |  |  | GG | 0.64 | 4.8x10^-3^ |
| rs1626783 | 2 | 0.34 | AA | ref. | ref. |
|  |  |  | AC | 1.3 | 6.7x10^-2^ |
|  |  |  | CC | 1.6 | 4.8x10^-3^ |
| rs4314631 | 8 | 0.37 | CC | ref. | ref. |
|  |  |  | CT | 0.83 | 0.13 |
|  |  |  | TT | 0.62 | 4.9x10^-3^ |
| rs4862655 | 4 | 0.46 | CC | ref. | ref. |
|  |  |  | CT | 0.85 | 0.24 |
|  |  |  | TT | 0.65 | 4.9x10^-3^ |
| rs2852192 | 11 | 0.58 | CC | ref. | ref. |
|  |  |  | CG | 0.89 | 0.30 |
|  |  |  | GG | 0.59 | 4.9x10^-3^ |
| rs2301826 | 15 | 0.47 | CC | ref. | ref. |
|  |  |  | CT | 0.77 | 2.8x10^-2^ |
|  |  |  | TT | 0.51 | 4.9x10^-3^ |
| rs935613 | 2 | 0.49 | CC | ref. | ref. |
|  |  |  | CT | 1.0 | 0.83 |
|  |  |  | TT | 0.60 | 4.9x10^-3^ |
| rs4833906 | 4 | 0.32 | AA | ref. | ref. |
|  |  |  | AT | 0.55 | 9.8x10^-3^ |
|  |  |  | TT | 0.53 | 4.9x10^-3^ |
| rs4339575 | 7 | 0.27 | AA | ref. | ref. |
|  |  |  | AG | 0.88 | 0.29 |
|  |  |  | GG | 0.65 | 4.9x10^-3^ |

Abbreviations: SNP, single nucleotide polymorphism; PSN, peripheral sensory neuropathy; F_ST_, fixation index; OR, odds ratio.

**Supplemental Table 5. List of SNPs with Greatest Association between Genotype and Vertigo Phenotype (p-value < 5x10^-3^)**

| SNP | chromosome | F_ST_ | Genotype | Vertigo Yes vs. No | |
| --- | --- | --- | --- | --- | --- |
|  |  |  |  | OR | p-value |
| rs2297771 | 9 | 0.38 | AA | ref. | ref. |
|  |  |  | AG | 0.057 | 6.4x10^-6^ |
|  |  |  | GG | 0.098 | 3.0x10^-5^ |
| rs3777909 | 6 | 0.27 | AA | ref. | ref. |
|  |  |  | AG | 0.14 | 6.6x10^-6^ |
|  |  |  | GG | 0.21 | 3.1x10^-5^ |
| rs28516482 | 5 | 0.26 | AA | ref. | ref. |
|  |  |  | AG | 0.13 | 1.4x10^-3^ |
|  |  |  | GG | 0.091 | 5.0x10^-5^ |
| rs56819906 | 11 | 0.34 | AA | ref. | ref. |
|  |  |  | AG | 0.19 | 1.5x10^-3^ |
|  |  |  | GG | 0.14 | 7.6x10^-5^ |
| rs663398 | 1 | 0.26 | AA | ref. | ref. |
|  |  |  | AG | 0.88 | 0.68 |
|  |  |  | GG | 7.5 | 1.2x10^-4^ |
| rs73626678 | 11 | 0.33 | CC | ref. | ref. |
|  |  |  | CT | 0.24 | 1.1x10^-3^ |
|  |  |  | TT | 0.21 | 1.8x10^-4^ |
| rs308442 | 4 | 0.57 | AA | ref. | ref. |
|  |  |  | AT | 0.33 | 1.2x10^-3^ |
|  |  |  | TT | 0.27 | 2.0x10^-4^ |
| rs28405640 | 14 | 0.37 | CC | ref. | ref. |
|  |  |  | CG | 1.9 | 3.7x10^-2^ |
|  |  |  | GG | 4.0 | 2.6x10^-4^ |
| rs7704574 | 5 | 0.58 | AA | ref. | ref. |
|  |  |  | AC | 0.64 | 0.19 |
|  |  |  | CC | 0.24 | 3.4x10^-4^ |
| rs167428 | 4 | 0.53 | CC | ref. | ref. |
|  |  |  | CT | 0.42 | 1.8x10^-2^ |
|  |  |  | TT | 0.26 | 4.2x10^-4^ |
| rs9469327 | 6 | 0.47 | CC | ref. | ref. |
|  |  |  | CT | 1.6 | 0.12 |
|  |  |  | TT | 4.1 | 5.4x10^-4^ |
| rs7987832 | 13 | 0.41 | AA | ref. | ref. |
|  |  |  | AG | 0.41 | 5.0x10^-3^ |
|  |  |  | GG | 0.29 | 5.5x10^-4^ |
| rs1561398 | 5 | 0.44 | CC | ref. | ref. |
|  |  |  | CT | 1.1 | 0.82 |
|  |  |  | TT | 3.4 | 5.9x10^-4^ |
| rs289802 | 15 | 0.62 | AA | ref. | ref. |
|  |  |  | AG | 0.090 | 9.9x10^-4^ |
|  |  |  | GG | 0.091 | 6.0x10^-4^ |
| rs12890652 | 14 | 0.29 | CC | ref. | ref. |
|  |  |  | CT | 0.33 | 3.4x10^-4^ |
|  |  |  | TT | 0.32 | 6.0x10^-4^ |
| rs3924985 | 10 | 0.33 | AA | ref. | ref. |
|  |  |  | AG | 0.16 | 1.8x10^-3^ |
|  |  |  | GG | 0.16 | 6.4x10^-4^ |
| rs2716578 | 16 | 0.41 | CC | ref. | ref. |
|  |  |  | CG | 0.24 | 6.2x10^-3^ |
|  |  |  | GG | 0.19 | 7.4x10^-4^ |
| rs6789962 | 3 | 0.42 | CC | ref. | ref. |
|  |  |  | CT | 0.11 | 4.8x10^-4^ |
|  |  |  | TT | 0.14 | 7.5x10^-4^ |
| rs8108374 | 19 | 0.32 | AA | ref. | ref. |
|  |  |  | AC | 0.48 | 9.5x10^-3^ |
|  |  |  | CC | 0.28 | 9.2x10^-4^ |
| rs2603749 | 12 | 0.50 | CC | ref. | ref. |
|  |  |  | CT | 0.10 | 5.7x10^-4^ |
|  |  |  | TT | 0.14 | 9.4x10^-4^ |
| rs6871601 | 5 | 0.28 | CC | ref. | ref. |
|  |  |  | CG | 0.52 | 6.8x10^-2^ |
|  |  |  | GG | 0.29 | 9.5x10^-4^ |
| rs7297582 | 12 | 0.26 | CC | ref. | ref. |
|  |  |  | CT | 1.4 | 0.27 |
|  |  |  | TT | 3.3 | 9.8x10^-4^ |
| rs2976230 | 17 | 0.29 | AA | ref. | ref. |
|  |  |  | AG | 1.1 | 0.70 |
|  |  |  | GG | 3.7 | 1.0x10^-3^ |
| rs73870865 | 3 | 0.33 | AA | ref. | ref. |
|  |  |  | AG | 0.063 | 1.2x10^-3^ |
|  |  |  | GG | 0.068 | 1.0x10^-3^ |
| rs7560799 | 2 | 0.28 | CC | ref. | ref. |
|  |  |  | CT | 0.31 | 2.6x10^-3^ |
|  |  |  | TT | 0.29 | 1.1x10^-3^ |
| rs1076743 | 6 | 0.26 | CC | ref. | ref. |
|  |  |  | CG | 2.2 | 7.5x10^-3^ |
|  |  |  | GG | 3.6 | 1.1x10^-3^ |
| rs6704593 | 2 | 0.27 | AA | ref. | ref. |
|  |  |  | AG | 0.31 | 1.6x10^-4^ |
|  |  |  | GG | 0.34 | 1.1x10^-3^ |
| rs56218597 | 16 | 0.42 | CC | ref. | ref. |
|  |  |  | CT | 0.30 | 1.0x10^-2^ |
|  |  |  | TT | 0.23 | 1.2x10^-3^ |
| rs11757702 | 6 | 0.47 | AA | ref. | ref. |
|  |  |  | AG | 1.1 | 0.79 |
|  |  |  | GG | 5.7 | 1.2x10^-3^ |
| rs6767856 | 3 | 0.36 | AA | ref. | ref. |
|  |  |  | AG | 4.3 | 4.1x10^-3^ |
|  |  |  | GG | 5.6 | 1.2x10^-3^ |
| rs112192561 | 6 | 0.29 | AA | ref. | ref. |
|  |  |  | AG | 0.41 | 6.6x10^-2^ |
|  |  |  | GG | 0.21 | 1.4x10^-3^ |
| rs932917 | 10 | 0.29 | AA | ref. | ref. |
|  |  |  | AC | 0.37 | 3.1x10^-2^ |
|  |  |  | CC | 0.23 | 1.4x10^-3^ |
| rs2004197 | 6 | 0.28 | AA | ref. | ref. |
|  |  |  | AC | 0.72 | 0.25 |
|  |  |  | CC | 0.26 | 1.5x10^-3^ |
| rs4851000 | 2 | 0.34 | CC | ref. | ref. |
|  |  |  | CT | 0.25 | 2.0x10^-5^ |
|  |  |  | TT | 0.36 | 1.6x10^-3^ |
| rs4440991 | 11 | 0.53 | AA | ref. | ref. |
|  |  |  | AT | 0.48 | 6.2x10^-2^ |
|  |  |  | TT | 0.28 | 1.6x10^-3^ |
| rs58824895 | 2 | 0.33 | CC | ref. | ref. |
|  |  |  | CT | 1.1 | 0.80 |
|  |  |  | TT | 6.4 | 1.7x10^-3^ |
| rs9467046 | 6 | 0.32 | AA | ref. | ref. |
|  |  |  | AG | 0.59 | 6.2x10^-2^ |
|  |  |  | GG | 0.29 | 1.8x10^-3^ |
| rs4529254 | 5 | 0.30 | GG | ref. | ref. |
|  |  |  | GT | 2.4 | 4.4x10^-3^ |
|  |  |  | TT | 3.5 | 1.8x10^-3^ |
| rs1067399 | 2 | 0.31 | AA | ref. | ref. |
|  |  |  | AG | 0.39 | 5.2x10^-3^ |
|  |  |  | GG | 0.34 | 2.3x10^-3^ |
| rs948421 | 8 | 0.52 | CC | ref. | ref. |
|  |  |  | CT | 0.59 | 8.1x10^-2^ |
|  |  |  | TT | 0.31 | 2.3x10^-3^ |
| rs7129085 | 11 | 0.31 | GG | ref. | ref. |
|  |  |  | GT | 4.0 | 1.9x10^-2^ |
|  |  |  | TT | 6.2 | 2.5x10^-3^ |
| rs564913 | 17 | 0.44 | AA | ref. | ref. |
|  |  |  | AC | 0.72 | 0.29 |
|  |  |  | CC | 0.30 | 2.6x10^-3^ |
| rs2289500 | 3 | 0.41 | CC | ref. | ref. |
|  |  |  | CG | 1.0 | 0.88 |
|  |  |  | GG | 2.8 | 2.7x10^-3^ |
| rs6578259 | 11 | 0.43 | AA | ref. | ref. |
|  |  |  | AT | 0.62 | 0.12 |
|  |  |  | TT | 0.32 | 2.8x10^-3^ |
| rs7432556 | 3 | 0.29 | CC | ref. | ref. |
|  |  |  | CT | 1.8 | 4.7x10^-2^ |
|  |  |  | TT | 3.0 | 2.9x10^-3^ |
| rs9913445 | 17 | 0.54 | CC | ref. | ref. |
|  |  |  | CT | 0.73 | 0.32 |
|  |  |  | TT | 0.28 | 2.9x10^-3^ |
| rs1374795 | 3 | 0.33 | AA | ref. | ref. |
|  |  |  | AG | 0.26 | 7.2x10^-3^ |
|  |  |  | GG | 0.25 | 3.1x10^-3^ |
| rs11788775 | 9 | 0.25 | AA | ref. | ref. |
|  |  |  | AG | 0.34 | 2.5x10^-3^ |
|  |  |  | GG | 0.35 | 3.2x10^-3^ |
| rs7561569 | 2 | 0.40 | AA | ref. | ref. |
|  |  |  | AC | 1.9 | 3.9x10^-2^ |
|  |  |  | CC | 3.1 | 3.2x10^-3^ |
| rs7897357 | 10 | 0.47 | CC | ref. | ref. |
|  |  |  | CT | 1.4 | 0.24 |
|  |  |  | TT | 2.9 | 3.4x10^-3^ |
| rs7191766 | 16 | 0.41 | AA | ref. | ref. |
|  |  |  | AG | 0.51 | 6.9x10^-2^ |
|  |  |  | GG | 0.33 | 3.5x10^-3^ |
| rs8028552 | 15 | 0.28 | CC | ref. | ref. |
|  |  |  | CT | 0.45 | 7.2x10^-2^ |
|  |  |  | TT | 0.28 | 3.5x10^-3^ |
| rs2346747 | 3 | 0.40 | AA | ref. | ref. |
|  |  |  | AG | 0.70 | 0.29 |
|  |  |  | GG | 0.33 | 3.6x10^-3^ |
| rs2945905 | 8 | 0.29 | GG | ref. | ref. |
|  |  |  | GT | 0.31 | 1.5x10^-2^ |
|  |  |  | TT | 0.24 | 3.8x10^-3^ |
| rs12423277 | 12 | 0.42 | AA | ref. | ref. |
|  |  |  | AC | 1.9 | 9.2x10^-2^ |
|  |  |  | CC | 3.1 | 3.9x10^-3^ |
| rs673253 | 1 | 0.29 | CC | ref. | ref. |
|  |  |  | CT | 1.7 | 0.10 |
|  |  |  | TT | 2.9 | 4.0x10^-3^ |
| rs10786134 | 10 | 0.32 | AA | ref. | ref. |
|  |  |  | AC | 1.0 | 0.87 |
|  |  |  | CC | 0.20 | 4.0x10^-3^ |
| rs7048572 | 9 | 0.45 | CC | ref. | ref. |
|  |  |  | CT | 0.45 | 5.8x10^-2^ |
|  |  |  | TT | 0.30 | 4.1x10^-3^ |
| rs4888325 | 16 | 0.25 | AA | ref. | ref. |
|  |  |  | AC | 0.51 | 2.0x10^-2^ |
|  |  |  | CC | 0.34 | 4.1x10^-3^ |
| rs454182 | 6 | 0.37 | CC | ref. | ref. |
|  |  |  | CG | 41 | 8.0x10^-3^ |
|  |  |  | GG | 58 | 4.1x10^-3^ |
| rs9471070 | 6 | 0.54 | CC | ref. | ref. |
|  |  |  | CT | 0.22 | 2.0x10^-2^ |
|  |  |  | TT | 0.17 | 4.2x10^-3^ |
| rs4131218 | 2 | 0.60 | AA | ref. | ref. |
|  |  |  | AG | 0.42 | 9.3x10^-3^ |
|  |  |  | GG | 0.36 | 4.2x10^-3^ |
| rs10206472 | 2 | 0.37 | CC | ref. | ref. |
|  |  |  | CG | 1.6 | 0.14 |
|  |  |  | GG | 2.9 | 4.2x10^-3^ |
| rs7990359 | 13 | 0.28 | AA | ref. | ref. |
|  |  |  | AG | 0.86 | 0.65 |
|  |  |  | GG | 7.1 | 4.3x10^-3^ |
| rs62168405 | 2 | 0.27 | CC | ref. | ref. |
|  |  |  | CT | 0.50 | 5.5x10^-2^ |
|  |  |  | TT | 0.35 | 4.3x10^-3^ |
| rs1085495 | 2 | 0.29 | AA | ref. | ref. |
|  |  |  | AG | 0.38 | 9.7x10^-3^ |
|  |  |  | GG | 0.35 | 4.4x10^-3^ |
| rs11834161 | 12 | 0.25 | AA | ref. | ref. |
|  |  |  | AC | 0.095 | 7.4x10^-4^ |
|  |  |  | CC | 0.16 | 4.4x10^-3^ |
| rs9378357 | 6 | 0.36 | GG | ref. | ref. |
|  |  |  | GT | 0.57 | 0.19 |
|  |  |  | TT | 6.1 | 4.5x10^-3^ |
| rs4464929 | 8 | 0.35 | CC | ref. | ref. |
|  |  |  | CG | 0.84 | 0.52 |
|  |  |  | GG | 0.23 | 4.6x10^-3^ |
| rs34382810 | 12 | 0.28 | AA | ref. | ref. |
|  |  |  | AC | 1.3 | 0.41 |
|  |  |  | CC | 2.7 | 4.6x10^-3^ |
| rs9487668 | 6 | 0.49 | AA | ref. | ref. |
|  |  |  | AG | 0.38 | 2.5x10^-2^ |
|  |  |  | GG | 0.32 | 4.7x10^-3^ |
| rs2028554 | 8 | 0.32 | GG | ref. | ref. |
|  |  |  | GT | 0.63 | 0.11 |
|  |  |  | TT | 0.32 | 4.7x10^-3^ |
| rs11604893 | 11 | 0.46 | CC | ref. | ref. |
|  |  |  | CT | 0.46 | 6.5x10^-3^ |
|  |  |  | TT | 0.34 | 4.7x10^-3^ |
| rs4953598 | 2 | 0.27 | CC | ref. | ref. |
|  |  |  | CT | 2.6 | 2.0x10^-3^ |
|  |  |  | TT | 3.1 | 4.7x10^-3^ |
| rs7742724 | 6 | 0.39 | AA | ref. | ref. |
|  |  |  | AG | 0.31 | 2.9x10^-3^ |
|  |  |  | GG | 0.35 | 4.8x10^-3^ |
| rs11720410 | 3 | 0.28 | CC | ref. | ref. |
|  |  |  | CT | 0.52 | 8.9x10^-2^ |
|  |  |  | TT | 0.33 | 5.0x10^-3^ |

Abbreviations: SNP, single nucleotide polymorphism; F_ST_, fixation index; OR, odds ratio.


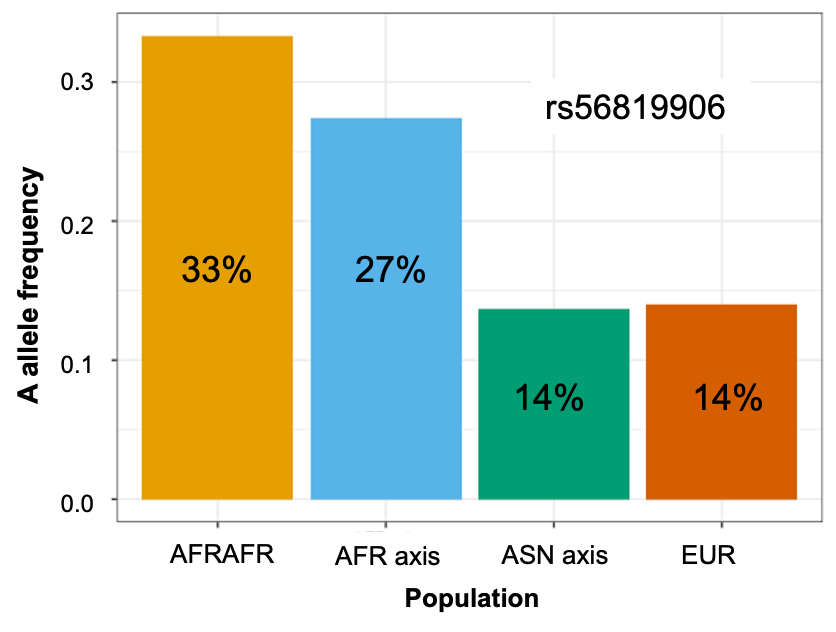
A)


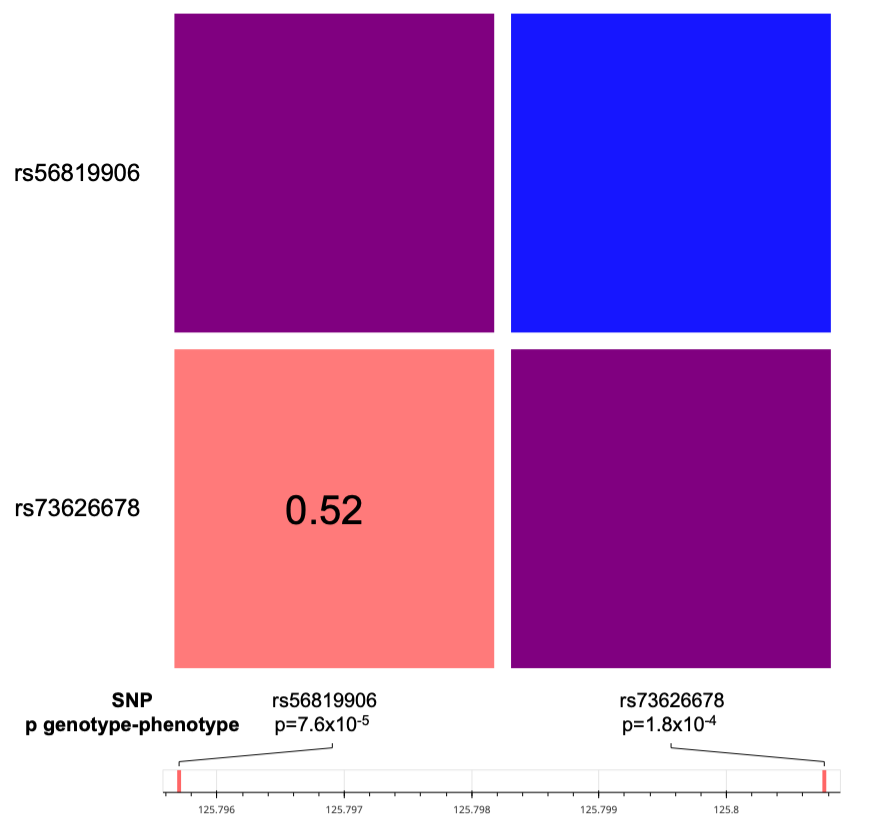


B)


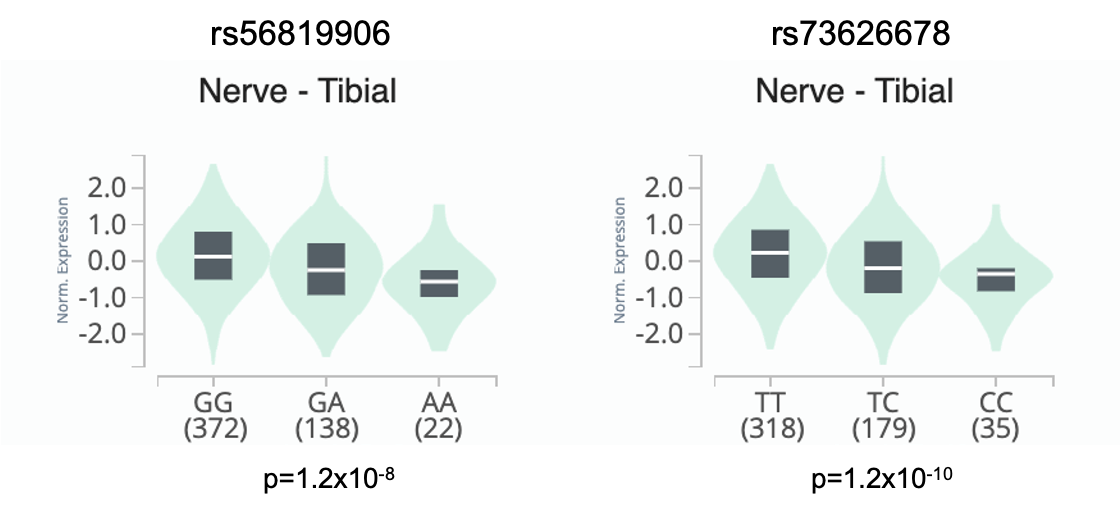


C)

**Supplemental Figure 3. eQTLs for DDX25 in Nerve Tibial Tissue are Associated with Vertigo. A,** A allele frequency for rs56819906 in testicular cancer survivors for AFRAFR, AFR axis, ASN axis, and EUR populations. **B,** Heatmap matrix of pairwise linkage disequilibrium statistics (R^2^ labeled) for eQTLs for DDX25 with association between SNP genotype and vertigo phenotype (p < 1x10^-3^ for all). **C,** Normalized gene expression and p-value in GTEx for eQTLs for DDX25 in nerve tibial tissue. Figure 3B was adapted from NCI LDmatrix. Figure 3C was generated using GTEX v8 data from the GTEx Portal between 10/2023 and 12/2023, with visualization created in 05/2024.
